# Construction of a Potential Breast Cancer-Related miRNA-mRNA Regulatory Network

**DOI:** 10.1155/2020/6149174

**Published:** 2020-11-04

**Authors:** Xinhong Liu, Feng Chen, Fang Tan, Fang Li, Ruokun Yi, Dingyi Yang, Xin Zhao

**Affiliations:** ^1^Chongqing Collaborative Innovation Center for Functional Food, Chongqing Engineering Research Center of Functional Food, Chongqing Engineering Laboratory for Research and Development of Functional Food, Chongqing University of Education, Chongqing 400067, China; ^2^College of Biological and Chemical Engineering, Chongqing University of Education, Chongqing 400067, China; ^3^Prehospital Emergency Department, Chongqing University Central Hospital, Chongqing Emergency Medical Center, Chongqing 400014, China; ^4^Department of Public Health, Our Lady of Fatima University, Valenzuela 838, Philippines; ^5^Key Laboratory for Biorheological Science and Technology of Ministry of Education (Chongqing University), Chongqing University Cancer Hospital & Chongqing Cancer Institute & Chongqing Cancer Hospital, Chongqing 400044, China

## Abstract

**Background:**

Breast cancer is a malignant tumor that occurs in the epithelial tissue of the breast gland and has become the most common malignancy in women. The regulation of the expression of related genes by microRNA (miRNA) plays an important role in breast cancer. We constructed a comprehensive breast cancer-miRNA-gene interaction map.

**Methods:**

Three miRNA microarray datasets (GSE26659, GSE45666, and GSE58210) were obtained from the GEO database. Then, the R software “LIMMA” package was used to identify differential expression analysis. Potential transcription factors and target genes of screened differentially expressed miRNAs (DE-miRNAs) were predicted. The BRCA GE-mRNA datasets (GSE109169 and GSE139038) were downloaded from the GEO database for identifying differentially expressed genes (DE-genes). Next, GO annotation and KEGG pathway enrichment analysis were conducted. A PPI network was then established, and hub genes were identified via Cytoscape software. The expression and prognostic roles of hub genes were further evaluated.

**Results:**

We found 6 upregulated differentially expressed- (DE-) miRNAs and 18 downregulated DE-miRNAs by analyzing 3 Gene Expression Omnibus databases, and we predicted the upstream transcription factors and downstream target genes for these DE-miRNAs. Then, we used the GEO database to perform differential analysis on breast cancer mRNA and obtained differentially expressed mRNA. We found 10 hub genes of upregulated DE-miRNAs and 10 hub genes of downregulated DE-miRNAs through interaction analysis.

**Conclusions:**

In this study, we have performed an integrated bioinformatics analysis to construct a more comprehensive BRCA-miRNA-gene network and provide new targets and research directions for the treatment and prognosis of BRCA.

## 1. Introduction

Breast cancer (BRCA) is the most common malignancy in women, with a significantly increasing incidence during the reproductive years [1]. BRCA is a primary cause of disease and death for women worldwide, [2, 3], and most BRCA-related deaths are due to metastasis [4]. However, because breast cancer is a heterogeneous tumor, there are great differences in histomorphology, immunophenotype, biological behavior, and treatment response. Patients with the same tumor, node, and metastasis (TNM) stage in traditional pathology may have very different responses to clinical treatment and prognosis [5].

The death rates from breast cancer continue to decrease as a result of the increased utilization of mammography. However, mammography is not a definitive early screening tool due to its limited sensitivity and specificity [6]. The pathogenesis of BRCA currently remains unclear, but it is considered to begin with alterations at the genomic level [7]. Therefore, to find and develop effective BRCA diagnosis and treatment methods, the current trend is to identify new targets and treatment strategies through the mining of big data [8].

Microribonucleic acids (miRNAs) are small noncoding RNAs that function as guide molecules in RNA silencing. Targeting most protein-coding transcripts, miRNAs are involved in nearly all developmental and pathological processes in animals [9, 10], such as cell proliferation [11], migration [12, 13], apoptosis [14], autophagy [15, 16], and energy homeostasis [17]. Misregulation of miRNA expression can cause many diseases, such as osteoporosis [18], diabetes [19], obesity [20], cancer [21], and nervous system disorders [22–24].

MiRNAs also closely function in the onset and progression of BRCA. For example, miR-218 and miR-129 regulate breast cancer progression by targeting lamins [25]. Anti-miR-203 suppresses estrogen receptor- (ER-) positive breast cancer growth and stemness by targeting SOCS3 [26]. The miR-638-mediated regulation of BRCA1 affects DNA repair and sensitivity to ultraviolet (UV) light and cisplatin in triple-negative breast cancer [27]. miR-490-3p inhibits the growth and invasiveness in triple-negative breast cancer by repressing the expression of TNKS2 [28]. miR-31 inhibits migration and invasion by targeting SATB2 in triple-negative breast cancer [29]. miRNA-215-5p suppresses the aggressiveness of breast cancer cells by targeting Sox9 [30].

Although there are a large number of reports describing the verification of the function of single miRNA in BRCA, a systematic and comprehensive analysis of the miRNA-mRNA regulatory network of BRCA clinical samples does not exist. The construction of a miRNA-mRNA regulatory network is necessary to gain a deeper understanding of the molecular mechanism of BRCA. Through integrated bioinformatics analysis, targets with research value and potential effects can be unearthed. At present, there have been many studies using bioinformatics methods to construct a disease miRNA-mRNA regulatory network. For example, Bardou et al. [31] constructed a miRNA-mRNA regulatory network in colorectal cancer and Pathan et al. [32] constructed a glioblastoma multiforme-related miRNA-mRNA regulatory network.

Herein, we first screened several miRNAs (DE-miRNAs) in BRCA tissues that were differentially expressed compared with normal tissues by analyzing the GSE26659, GSE45666, and GSE58210 datasets downloaded from the Gene Expression Omnibus (GEO) database. FunRich was employed to predict the transcription factors upstream of the DE-miRNAs. Next, the miRNet database was introduced to predict potential target genes. Then, DE-mRNAs between BRCA tissues and normal tissues were obtained by analyzing the GSE109169 and GSE139038 datasets downloaded from the GEO database. Subsequently, gene ontology (GO) functional annotation and Kyoto Encyclopedia of Genes and Genomes (KEGG) pathway enrichment analysis were conducted by the Enrichr database.

Hub genes were identified with the help of Cytoscape (cytoHubba) software, and the expression levels and prognostic roles of these hub genes were further determined by the Gene Expression Profiling Interactive Analysis (GEPIA) database. Finally, a potential miRNA-mRNA regulatory network contributing to the onset and progression of BRCA was successfully established.

## 2. Materials and Methods

### 2.1. Microarray Dataset

The bioinformatics analysis part of this study followed the methods of Pathan et al. [32]. During the initial stage, we searched for the expression database of BRCA-related miRNAs in the National Center for Biotechnology Information (NCBI) GEO database (https://www.ncbi.nlm.nih.gov/geo/). We only selected databases derived from human BRCA patients and normal samples that contained more than 50 samples. Animal and cell line source data were excluded. Finally, microarray datasets GSE26659, GSE45666, GSE58210, GSE109169, and GSE139038 met the abovementioned criteria and were selected for our subsequent analysis.

Dataset GSE26659 was based on the platform of GPL8227 (Agilent-019118 Human miRNA Microarray 2.0 G4470B (miRNA ID version)) and contained 77 frozen tumor specimens and 17 normal tissues from mammoplastic reductions. Dataset GSE45666 was based on the platform of GPL14767 (Agilent-021827 Human miRNA Microarray G4470C (Feature Number version)) and contained 101 breast tumors and 15 adjacent breast normal tissue samples. Dataset GSE58210 was based on the platform of GPL10656 (Agilent-029297 Human miRNA Microarray v14 Rev.2 (miRNA ID version)) and contained 283 breast tumors and 25 adjacent breast normal tissue samples. Dataset GSE109169 was based on the platform of GPL5175 (Affymetrix Human Exon 1.0 ST Array (transcript (gene) version)) and contained 25 breast tumors and 25 adjacent breast normal tissue samples. Dataset GSE139038 was based on the platform of GPL27630 (Print_1437 (Block_Column_Row IDs)) and contained 41 breast tumors and 24 adjacent normal breast tissue samples.

### 2.2. Identification of Differentially Expressed miRNAs (DE-miRNAs)

The R software “LIMMA” package was used to identify DE-miRNAs between BRCA tissues and normal tissues. We first used the GEO Convenient Converter to convert GEO original MINiML formatted family file(s). Then, we conducted a differential analysis of the converted files to obtain different DE-miRNAs.

To identify the greatest number of possible miRNAs that are involved in the pathogenesis of BRCA, we analyzed the GSE26659, GSE45666, and GSE58210 databases. The DE-miRNAs that commonly appeared in three differential expression analyses were finally selected for subsequent investigation. |Log_2_FC| > 1 and adj *P* < 0.05 were set as the thresholds for identifying DE-miRNAs. Volcano diagrams were produced by GraphPad Prism 7, and Venn diagrams were produced by online tools (http://jvenn.toulouse.inra.fr/app/example.html) [33].

### 2.3. Prediction of Potential Transcription Factors

The upstream transcription factors of DE-miRNAs were predicted by FunRich software, which is a stand-alone software tool mainly used for functional enrichment and interaction network analysis of genes and proteins [34]. We input upregulated and downregulated DE-miRNAs to obtain their upstream transcription factors, and present the top ten transcription factors according to *P* value.

### 2.4. Prediction of Potential Target Genes of DE-miRNAs

The downstream target genes of DE-miRNAs were predicted by miRNet software, which is an easy-to-use web-based tool that offers statistical, visual, and network-based approaches to assist researchers in increasing their understanding of miRNA functions and regulatory mechanisms [35]. We input upregulated and downregulated DE-miRNAs to obtain their downstream target genes.

### 2.5. Identification of Differentially Expressed mRNAs (DE-Genes)

In order to improve the reliability of our target genes of screened DE-miRNAs, we downloaded the BRCA mRNA expression datasets GSE109169 and GSE139038 from the GEO database, analyzed the differentially expressed mRNAs, and intersected them with target genes to obtain the final candidate target genes. |Log_2_FC| > 1 and adj *P* < 0.05 were set as the thresholds for identifying DE-genes.

### 2.6. GO Annotation and KEGG Pathway Enrichment Analysis

The GO functional annotation and KEGG pathway enrichment analysis for the DE-genes were conducted by Enrichr, which is a comprehensive resource for curated gene sets and a search engine that accumulates biological knowledge for further biological discoveries (http://amp.pharm.mssm.edu/Enrichr/) [36]. We input upregulated and downregulated DE-genes, and conducted biological process (BP), cellular component (CC), and molecular function (MF) functional annotation and KEGG pathway enrichment analysis. The top ten results are presented according to *P* value, with *P* < 0.05 considered as statistically significant.

### 2.7. Establishment and Analysis of the Protein-Protein Interaction (PPI) Network

In order to better understand the relationship between candidate target genes, we constructed a PPI network using the STRING database, which is a database that is aimed at collecting and integrating this information by consolidating known and predicted protein-protein association data for a large number of organisms [37]. PPI node pairs with a combined score ≥ 0.4 were selected for further analysis.

The hub genes in the PPI network were identified according to degree using Cytoscape (cytoHubba) software (version 3.6.1), which is an open source software project for integrating biomolecular interaction networks with high-throughput expression data and other molecular states into a unified conceptual framework [38].

### 2.8. Validation of Hub Gene Expression Levels

The expression levels of 20 hub genes in BRCA patients and normal samples were verified by GEPIA, which is a web-based tool that delivers fast and customizable functionalities based on TCGA and Genotype Tissue Expression (GTEx) data and provides key interactive and customizable functions including differential expression analysis, profiling plotting, correlation analysis, patient survival analysis, similar gene detection, and dimensionality reduction analysis [39]. Hub genes with |log_2_FC| > 1 and *P* < 0.05 were considered as statistically significant.

### 2.9. Validation of Hub Gene Survival

The survival of 20 hub genes in BRCA patients was verified by GEPIA, and the survival results are presented in the form of diagrams. The hazards ratio is calculated based on the Cox PH Model; cutoff-high and cutoff-low are both 50%.

### 2.10. Oligonucleotide Synthesis and Plasmid Constructs

MiR-454 mimics, miR-454 inhibitors, miRNA NC, inhibitor NC, *PRNP* siRNA, and siRNA NC oligonucleotide primers were chemically synthesized (Gene Pharma, Shanghai, China). The complete coding sequence of *PRNP* was chemically synthesized and inserted between the XhoI and EcoRI restriction sites of the PI RES2-EGFP vector to construct an overexpression vector of *PRNP*. The 1000 bp nucleotide sequence of the downstream noncoding region of PRNP was chemically synthesized and constructed between the XhoI and NotI restriction sites of the pSi-Check2 vector to construct the *PRNP* wild-type 3′UTR vector. We used a point mutation method to replace the bases at the binding position of miRNA-454 and *PRNP*, and constructed the *PRNP* mutant-type 3′UTR vector.

### 2.11. Cell Cultures

*Homo sapiens* breast cancer cell lines MDA-MB-231 and MCF-7 and breast cell line MCF-10A were purchased from the Cell Bank of the Chinese Academy of Sciences (Shanghai, China). These three cell lines were maintained according to the vendor's instructions. In brief, MDA-MB-231 cells were cultured in Leibovitz's L-15 Medium with 10% fetal bovine serum (FBS) and 1% penicillin-streptomycin (100 U/ml penicillin and 100 *μ*g/ml streptomycin). MCF-7 cells were cultured in MEM medium (add NaHCO_3_ 1.5 g/l, sodium pyruvate 0.11 g/l, and 0.01 mg/ml bovine insulin) (HyClone, Waltham, MA, USA) with 10% fetal bovine serum (FBS) and 1% penicillin-streptomycin. MCF-10A cells were cultured in the complete culture medium MEGM kit with 10% FBS and 1% penicillin-streptomycin. MCF-7 and MCF-10A cells were maintained at 37°C in a humidified incubator at 5% CO_2_, and MDA-MB-231 cells were maintained at 37°C in a humidified incubator at 100% air.

### 2.12. Cell Transfection and Luciferase Reporter Assay

The *PRNP* wild-type 3′UTR vector, *PRNP* mutant-type 3′UTR vector, miR-454 mimics, mimic NC, miR-454 inhibitors, and inhibitor NC were transfected in cells at 20 nM using the Lipofectamine 3000 Transfection Reagent (Invitrogen, Carlsbad, CA, USA) with serum-free media for 48 h per the manufacturer's instructions (the amount of plasmids is 2 *μ*g/well). The luciferase reporter assay was performed using the Dual-Luciferase Reporter Assay System (Promega, Madison, WI, USA), and the relative luciferase activity was calculated after transfection for 48 h by normalizing the firefly luminescence to that of Renilla. Each experiment consisted of three technical replicates, and three independent biological replicates were performed. Oligonucleotide sequences are presented in Table 1.

### 2.13. Cell Proliferation Assay

The MTT cell proliferation assay (Solarbio, Beijing, China) was performed to analyze the function of *PRNP* on breast cancer cell proliferation. Briefly, the transfected MDA-MB-231 cells and MCF-7 cells were seeded into 96-well plates at a density of 2 × 10^4^ cells per well. The cells were cultured overnight at 37°C in a humidified incubator with 5% CO_2_ or 100% air. Then, an MTT reagent was added to each well and incubated for 4 h on the 1st day, 3rd day, 5th day, and 7th day. Afterwards, the absorbance was measured at 490 nm. All experiments were performed in triplicate.

### 2.14. Statistical Analysis

Most of the statistical analyses were performed using the bioinformatic tools mentioned above. When we conducted differential expression analysis, only genes or miRNAs with |log_2_FC| > 1 and *P* < 0.05 were considered as statistically significant. Cox *P* < 0.05 was considered as statistically significant for survival analysis.

## 3. Results

### 3.1. Identification of Candidate DE-miRNAs

**Figure 1:**
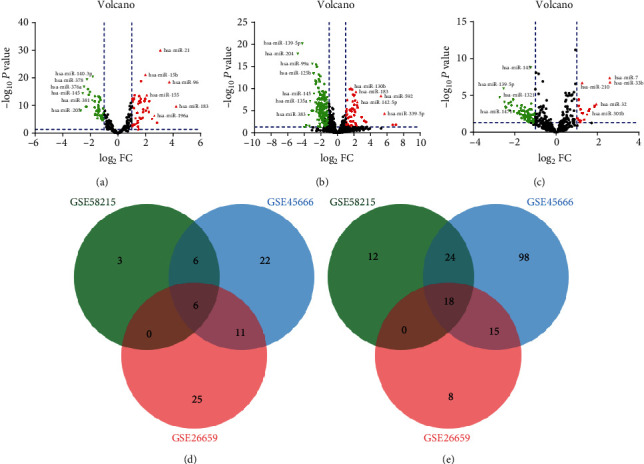
Identification of differentially expressed miRNAs (DE-miRNAs): (a) BRCA DE-miRNAs of the GSE62259 database; (b) BRCA DE-miRNAs of the GSE45666 database; (c) BRCA DE-miRNAs of the GSE58210 database; (d) intersection of the three GEO databases with upregulated DE-miRNAs; (e) intersection of the three GEO databases with downregulated DE-miRNAs.

By analyzing the three datasets, GSE26659, GSE45666, and GSE58210, we obtained the BRCA DE-miRNAs and present them in Figures 1(a)–1(c). Only the DE-miRNAs commonly appearing in the three sets were chosen as candidate DE-miRNAs, as shown in Figures 1(d) and 1(e). We found 6 upregulated DE-miRNAs (hsa-miR-7, hsa-miR-210, hsa-miR-96, hsa-miR-183, hsa-miR-141, and hsa-miR-454) and 18 downregulated DE-miRNAs (hsa-miR-378, hsa-miR-376a, hsa-miR-145, hsa-miR-202, hsa-miR-381, hsa-miR-601, hsa-miR-139-5p, hsa-miR-379, hsa-miR-205, hsa-miR-99a, hsa-miR-483-5p, hsa-miR-335, hsa-miR-370, hsa-miR-154, hsa-miR-126, hsa-miR-486-5p, hsa-miR-143, and hsa-miR-513a-5p), which are presented in Table S1.

### 3.2. Prediction of Upstream Transcription Factors of DE-miRNAs

**Figure 2:**
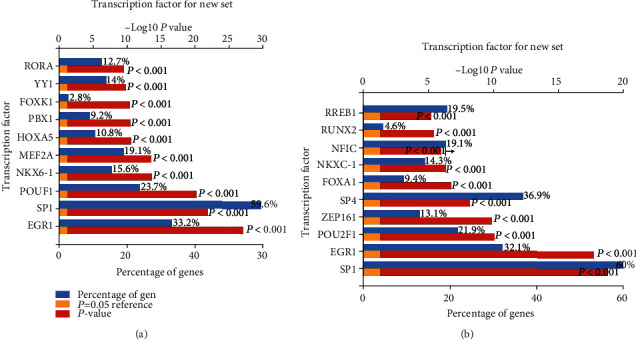
Predicted transcription factors of DE-miRNAs: (a) top ten upstream transcription factors of upregulated DE-miRNAs; (b) top ten upstream transcription factors of downregulated DE-miRNAs.

We predicted the upstream transcription factors of upregulated and downregulated DE-miRNAs through FunRich software, and the top 10 transcriptions are presented in Figures 2(a) and 2(b), respectively. For upregulated DE-miRNAs, the top 10 transcription factors were EGR1, SP1, POU2F1, NKX6-1, MEF2A, HOXA5, PBX1, FOXK1, YY1, and RORA. For downregulated DE-miRNAs, the top 10 transcription factors were SP1, EGR1, POU2F1, ZFP161, SP4, FOXA1, NKX6-1, NFIC, RUNX2, and RREB1.

### 3.3. Prediction of Downstream Target Genes of DE-miRNAs

**Figure 3:**
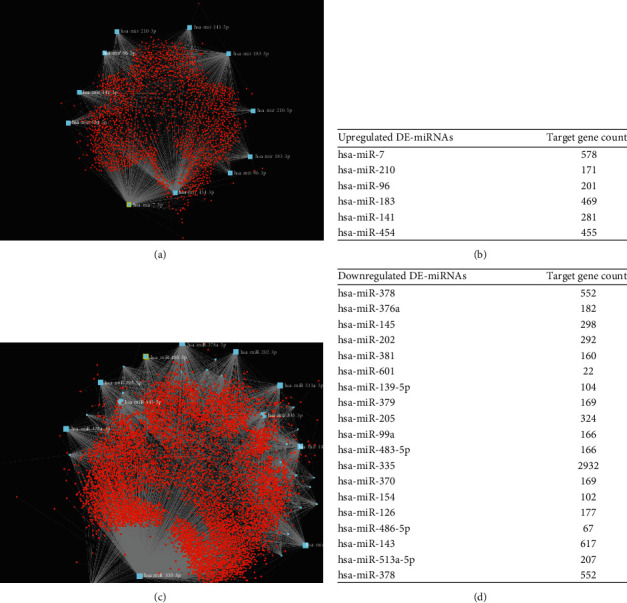
Potential target genes of DE-miRNAs predicted by databases: (a) upregulated miRNA-target gene network constructed using miRNet; (b) upregulated miRNA-target gene count; (c) downregulated miRNA-target gene network constructed using miRNet; (d) downregulated miRNA-target gene count.

We predicted the target genes of upregulated and downregulated DE-miRNAs through the miRNet database and present them in Table S2. We found 1920 target genes of upregulated DE-miRNAs and 5285 target genes of downregulated DE-miRNAs. In order to more intuitively show the relationship between DE-miRNAs and target genes, we plotted the DE-miRNA-target gene network (Figures 3(a) and 3(c)) and counted the target genes of different miRNAs (Figures 3(b) and 3(d)).

### 3.4. Identification of Candidate Target Genes

**Figure 4:**
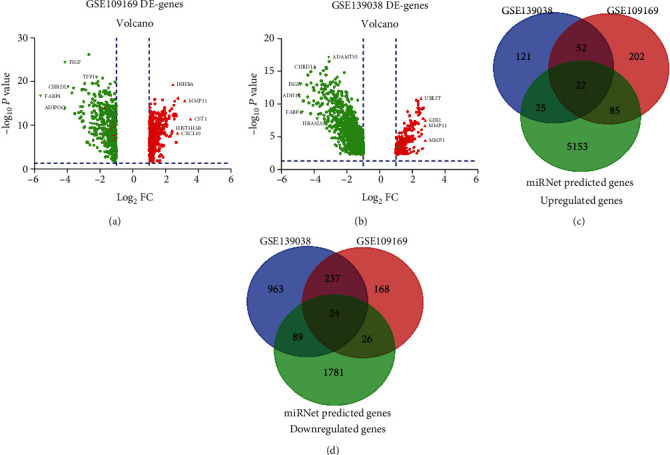
Identification of differentially expressed mRNAs (DE-genes): (a) BRCA DE-genes of the GSE109169 database; (b) BRCA DE-genes of the GSE139038 database; (c) intersection of downregulated miRNA-target genes and upregulated genes; (d) intersection of upregulated miRNA-target genes and downregulated genes.

In order to further increase the reliability of predicting target genes, we analyzed the two datasets GSE109169 and GSE139038, and obtained differentially expressed BRCA genes, which are presented in Figures 4(a) and 4(b), respectively. We intersected the analyzed DE-genes with the target genes predicted in the previous step to obtain the final candidate target genes. The data are presented in Figures 4(c) and 4(d) and Table S3.

We obtained 22 upregulated genes (*BUB1*, *GJB2*, *SPP1*, *CENPF*, *TTK*, *MMP11*, *MELK*, *MMP13*, *CTHRC1*, *E2F8*, *MMP1*, *OLR1*, *LMNB1*, *SQLE*, *RAD51*, *RACGAP1*, *COL10A1*, *HELLS*, *STAT1*, *CST1*, *CXCL9*, and *OAS2*) and 24 downregulated genes (*ABCA6*, *ABCD2*, *CAV1*, *CPE*, *CRIM1*, *CXCL12*, *DPYSL2*, *EGR1*, *FAT2*, *FBLN5*, *FOS*, *KLF4*, *LAMA2*, *MRAS*, *MYH11*, *NFIB*, *NR3C1*, *PRNP*, *RECK*, *SLIT3*, *TFPI*, *TGFBR2*, *TXNIP*, and *ZFHX4*).

### 3.5. Functional Annotation and Pathway Enrichment Analysis

**Figure 5:**
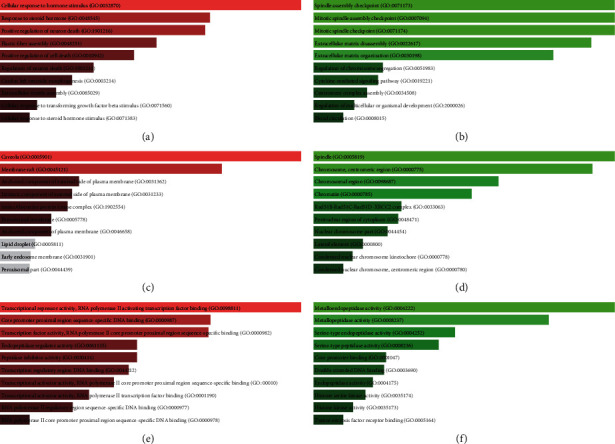
GO functional annotation of candidate target genes: (a) the top 10 enriched BP items of upregulated DE-miRNA candidate target genes; (b) the top 10 enriched BP items of downregulated DE-miRNA candidate target genes; (c) the top 10 enriched CC items of upregulated DE-miRNA candidate target genes; (d) the top 10 enriched CC items of downregulated DE-miRNA candidate target genes; (e) the top 10 enriched MF items of upregulated DE-miRNA candidate target genes; (f) the top 10 enriched MF items of downregulated DE-miRNA candidate target genes.

The Enrichr database was utilized to perform GO functional annotation and KEGG pathway enrichment analysis. The GO functional annotation included three categories, namely, biological process (BP), cellular component (CC), and molecular function (MF). The top 10 enriched GO items are listed in Figure 5.

The biological process analysis results show that the biological process of candidate target genes of upregulated DE-miRNAs is mainly concentrated in cellular response to hormonal stimulus, response to steroid hormones, and positive regulation of neuron death (Figure 5(a)), and the biological process of candidate target genes of downregulated DE-miRNAs is mainly concentrated in spindle assembly checkpoint, mitotic spindle assembly checkpoint, and mitotic spindle checkpoint (Figure 5(b)). The cellular component analysis results show that the cellular component of candidate target genes of upregulated DE-miRNAs is mainly concentrated in the caveola, membrane raft, and anchored component of the external side of the plasma membrane (Figure 5(c)), and the cellular component of candidate target genes of downregulated DE-miRNAs is mainly concentrated in the spindle, chromosome centromeric region, and chromosomal region (Figure 5(d)). The molecular function analysis results show that the molecular function of candidate target genes of upregulated DE-miRNAs is mainly concentrated in transcriptional repressor activity, RNA polymerase II activating transcription factor binding, and core promoter proximal region sequence-specific DNA binding (Figure 5(e)), and the molecular function of candidate target genes of downregulated DE-miRNAs is mainly concentrated in metalloendopeptidase activity, metallopeptidase activity, and serine-type endopeptidase activity (Figure 5(f)).

**Figure 6:**
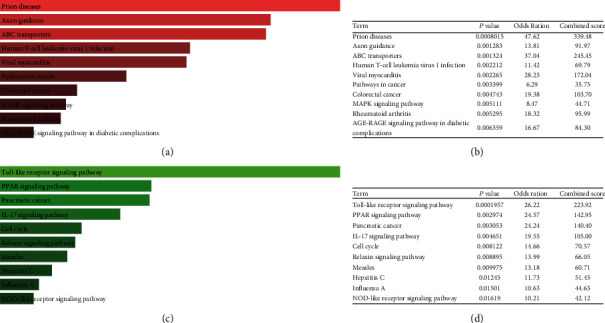
KEGG pathways of candidate target genes: (a, b) the top 10 enriched KEGG pathways for the candidate target genes of upregulated DE-miRNAs; (c, d) the top 10 enriched KEGG pathways for the candidate target genes of downregulated DE-miRNAs.

We further conducted a KEGG pathway enrichment analysis on target genes of DE-miRNAs, and the top 10 results are shown in Figure 6. Candidate target genes of upregulated DE-miRNAs were significantly enriched in Prion diseases, axon guidance, ABC transporters, human T-cell leukemia virus 1 infection, viral myocarditis, pathways in cancer, colorectal cancer, MAPK signaling pathway, rheumatoid arthritis, and the AGE-RAGE signaling pathway in diabetic complications (Figures 6(a) and 6(b)). Candidate target genes of downregulated DE-miRNAs were significantly enriched in the Toll-like receptor signaling pathway, PPAR signaling pathway, pancreatic cancer, interleukin- (IL-) 17 signaling pathway, cell cycle, relaxin signaling pathway, measles, hepatitis C, influenza A, and the NOD-like receptor (NLR) signaling pathway (Figures 6(c) and 6(d)).

### 3.6. Screen of Hub Genes

**Figure 7:**
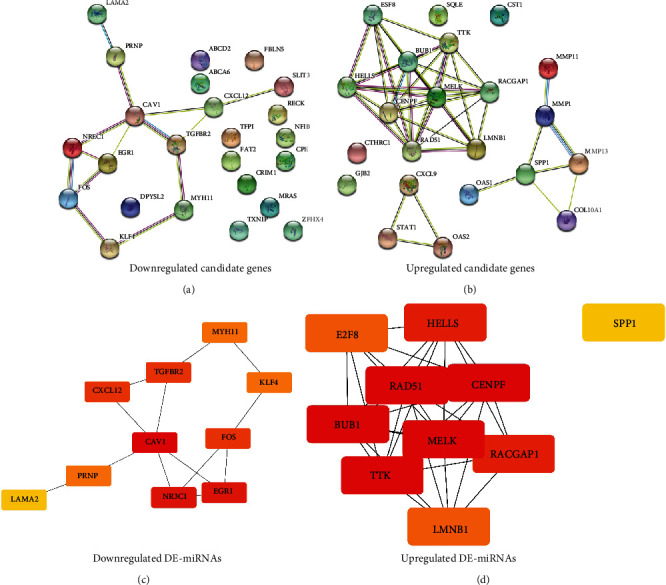
BRCA candidate target gene PPI network and hub gene network: (a) PPI network of downregulated candidate target genes; (b) PPI network of upregulated candidate target genes; (c) hub gene network of upregulated DE-miRNAs; (d) hub gene network of downregulated DE-miRNAs.

In order to better understand the relationship between these candidate target genes, we constructed a PPI network using the STRING database, and the results are shown in Figures 7(a) and 7(b). In order to find the hub genes in the PPI network, we downloaded the node pair Tab-Separated Values (TSV) files of the PPI network of downregulated candidate genes and upregulated candidate genes, entered them into the Cytoscape software, performed the calculations using the cytoHubba plug-in, and finally obtained the hub genes. The top 10 hub genes are presented in Figures 7(c) and 7(d).

**Figure 8:**
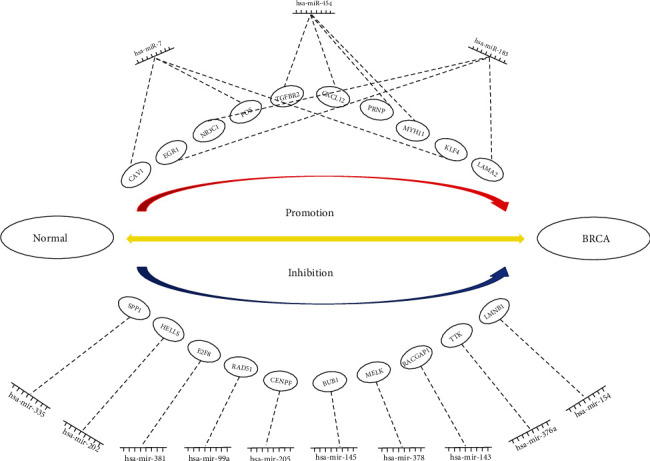
The miRNA-hub gene regulatory network in BRCA.

For the target genes of upregulated DE-miRNAs, the hub genes were *CAV1*, *EGR1*, *NR3C1*, *CXCL12*, *FOS*, *TGFBR2*, *PRNP*, *MYH11*, *KLF4*, and *LAMA2*. For the target genes of downregulated DE-miRNAs, the hub genes were *SPP1*, *HELLS*, *E2F8*, *RAD51*, *CENPF*, *BUB1*, *MELK*, *RACGAP1*, *TTK*, and *LMNB1*. Based on the previously predicted miRNA-mRNA binding and hub gene analysis, we finally constructed the candidate miRNA-hub gene regulatory network associated with BRCA and presented it in the form of a diagram (Figure 8).

### 3.7. Identification of Potential miRNA-mRNA Regulatory Pathways

**Figure 9:**
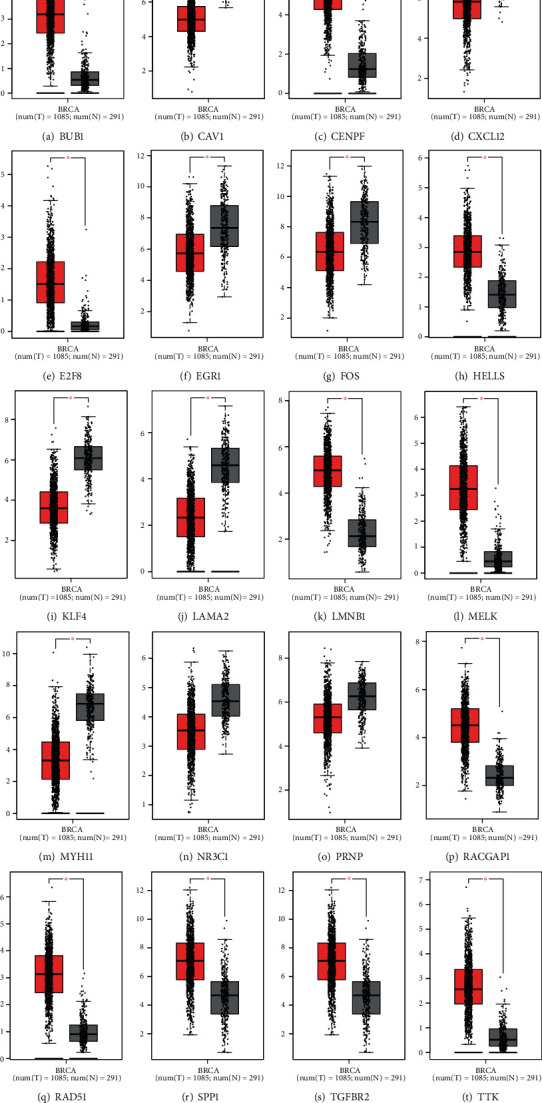
Expression of 20 hub genes in BRCA patients and normal samples. T = tumour; N = normal; *P* ≤ 0.05.

**Figure 10:**
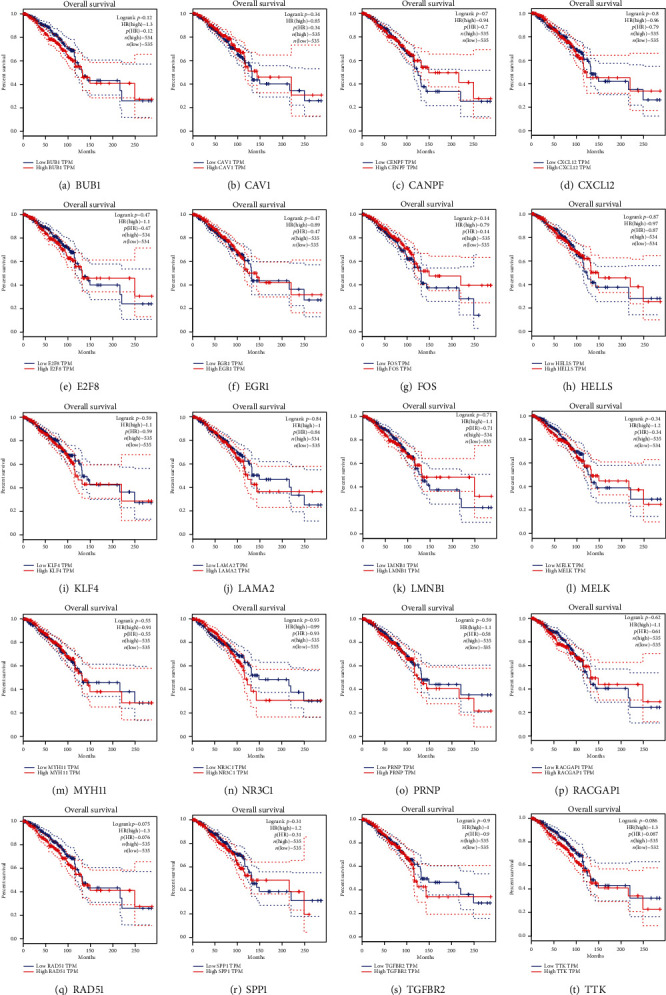
Survival of 20 hub genes in BRCA patients.

In order to further evaluate the function of hub genes in BRCA and the potential role of DE-miRNAs, we performed expression analysis and survival analysis on 20 hub genes through the GEPIA database, and the results are shown in Figures 9 and 10. Eight of the 10 hub genes of upregulated DE-miRNAs were markedly downregulated in BRCA tissues when compared with normal tissues. The expression levels of *NR3C1* and *PRNP* in BRCA samples also decreased compared with normal tissues, but the difference was not significant.

Marked upregulation in BRCA tissues was observed in 10 hub genes of downregulated DE-miRNAs when compared with normal tissues. In the hub genes of upregulated DE-miRNAs, samples with high expression of *FOS* had higher survival rates than samples with low expression, which showed a worse prognosis in BRCA. In the hub genes of downregulated DE-miRNAs, samples with high expression of *CENPF*, *E2FB*, *HELLS*, and *LMNB1* exhibited higher survival rates than samples with low expression, which showed a better prognosis in BRCA.

### 3.8. PRNP Is a Direct Target of miR-454

**Figure 11:**
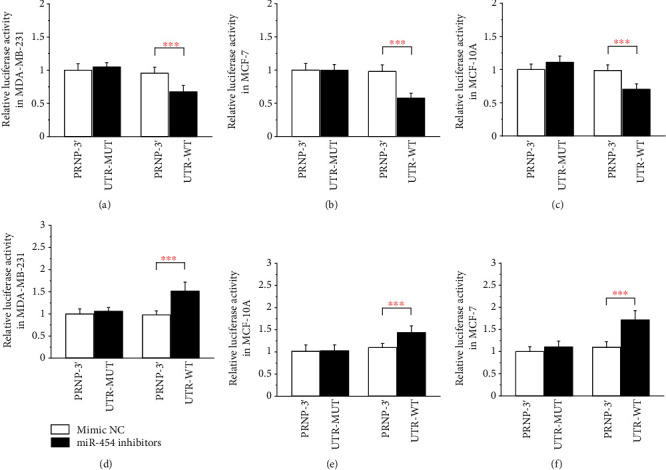
PRNP is a direct target of miR-454: (a) relative luciferase activity after transfection of MDA-MB-231 with miR-454 mimics; (b) relative luciferase activity after transfection of MCF-7 with miR-454 mimics; (c) relative luciferase activity after transfection of MCF-10A with miR-454 mimics; (d) relative luciferase activity after transfection of MDA-MB-231 with miR-454 inhibitors; (e) relative luciferase activity after transfection of MCF-7 with miR-454 inhibitors; (f) relative luciferase activity after transfection of MCF-10A with miR-454 inhibitors.

Previous studies have shown that miR-454 is an oncogene of breast cancer, promotes the onset of breast cancer, and is associated with a poor prognosis [40–42]. Related research shows that *PRNP* is an endoplasmic reticulum stress-regulated gene that could increase survival in breast cancers [43]. To further verify whether miRNA-454 can bind to endogenous *PRNP*, we performed dual luciferase reporter gene experiments in three cell lines. Cells were cotransfected with *PRNP*-3′UTR-WT and miR-454 mimics which resulted in a significantly reduced activity of the luciferase reporter gene, yet the luciferase activity was not significantly attenuated in the target region of the mutated *PRNP*-3′UTR-MUT construct. When cotransfected with *PRNP*-3′UTR-WT and miR-454 inhibitors, the activity of the luciferase reporter gene is significantly upregulated, but the *PRNP*-3′UTR-MUT construct has no such change (Figure 11).

### 3.9. PRNP Decreased the Proliferation of Breast Cancer Cells

**Figure 12:**
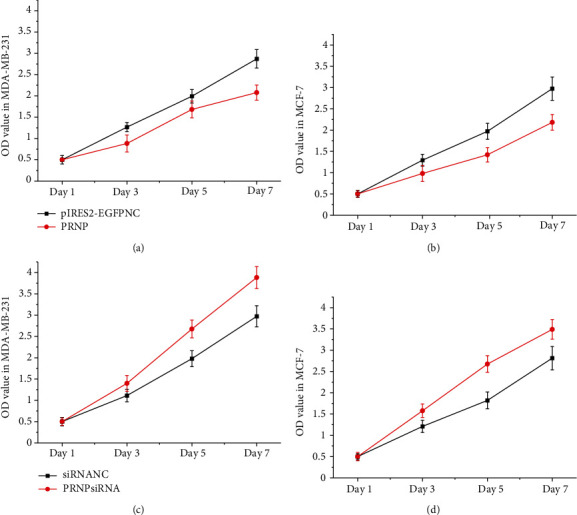
PRNP decreased the proliferation of breast cancer cells: (a) the proliferation of MDA-MB-231 cells after transfection of PRNP; (b) the proliferation of MCF-7 cells after transfection of PRNP; (c) the proliferation of MDA-MB-231 cells after transfection of PRNP siRNA; (d) the proliferation of MCF-7 cells after transfection of PRNP siRNA.

To examine the function of *PRNP* on the proliferation of breast cancer cells, *PRNP* was transfected to MDA-MB-231 and MCF-7 cells with Lipofectamine 3000. An MTT assay was performed to examine the proliferation. As shown in Figure 12, *PRNP* inhibited the proliferation of MDA-MB-231 and MCF-7 cells. Inhibition of the expression of PRNP promoted the proliferation of MDA-MB-231 and MCF-7 cells.

## 4. Discussion

Effective management of breast cancer depends on an early diagnosis and proper monitoring of response to therapy. However, these goals are difficult to achieve because of the lack of sensitive and specific biomarkers for early detection and for disease monitoring. Accumulating evidence in the past several years has highlighted the potential use of peripheral blood circulating nucleic acids such as miRNA in breast cancer diagnosis and prognosis, and for monitoring the response to anticancer therapy [44]. For example, miR-9, miR-200, and miR-10b can be used for early diagnosis of BRCA [45, 46]; miR-17-5p may serve as a novel predictor for breast cancer recurrence [47]; and miR-155 and miR-21 can be used as diagnostic markers for cell migration and invasion control [48, 49]. However, thus far, a comprehensive miRNA-gene regulatory network in BRCA has still not been created.

Through bioinformatics, we have constructed a BRCA-miRNA-gene regulatory network containing 13 DE-miRNAs and 20 hub genes. Some miRNAs confirmed by earlier studies can also be found in our network. For example, miR-335 inhibits the migration of BRCA cells through targeting oncoprotein c-Met [50]; miR-202 inhibits cell proliferation, invasion, and migration in BRCA by targeting the ROCK1 gene [51]; miR-381 inhibits BRCA cell proliferation, epithelial-to-mesenchymal transition, and metastasis by targeting CXCR4 [52]; miRNA-205 inhibits the proliferation and invasion of BRCA by regulating AMOT expression [53]; miR-145-5p suppresses BRCA progression by inhibiting SOX2 [54]; miR-378 mediates the metabolic shift in BRCA cells via the PGC-1*β*/ERR*γ* transcriptional pathway [55]; miR-143 inhibits the metastasis and invasion of BRCA cells [56]; miRNA-154 inhibits the growth and invasion of BRCA cells through targeting E2F5 [57]; and mir-183 promotes proliferation and migration in BRCA cell lines [58]. However, miR-99, miR-376a, and miR-7 have no direct functional verification studies in BRCA. The relationship between the abovementioned miRNAs that have been verified to function and our predicted target hub genes and BRCA has not been verified. This provides us with a new strategy for further research on BRCA-miRNA-genes.

When predicting transcription factors for DE-miRNA, we found that whether upregulated DE-miRNAs or downregulated DE-miRNAs exist, their top three transcription factors are SP1, EGR1, and POU2F1. SP1 is a zinc finger transcription factor that binds to GC-rich motifs of many promoters. The encoded protein is involved in many cellular processes, including cell differentiation, cell growth, apoptosis, immune responses, response to DNA damage, and chromatin remodeling. Moreover, a large number of experiments have proved that SP1 possesses an important role in the initiation of BRCA [59–62].

EGR1 belongs to the early growth response (EGR) factor family of C2H2-type zinc-finger proteins. It is a nuclear protein and functions as a transcriptional regulator. The products of the target genes it activates are required for differentiation and mitogenesis. Studies suggest that this is a gene that suppresses cancer, including BRCA [63]. POU2F1 has also been shown to be associated with the occurrence of multiple cancers [64, 65]. These findings suggest that the DE-miRNAs we mine may be regulated by these transcription factors, which in turn affects the occurrence of BRCA, and provides a direction for us to further study the function of DE-miRNAs.

Through the STRING database and Cytoscape software, we constructed a DE-mRNA interaction map and analyzed 10 hub genes for upregulated DE-miRNAs and 10 hub genes for downregulated DE-miRNAs. Then, we expressed these hub genes for analysis and survival analysis.

It was found that most hub genes had expression differences and survival differences in BRCA patients and normal samples. Eight of the 10 hub genes of upregulated DE-miRNAs were markedly downregulated in BRCA tissues when compared with normal tissues. We suspected that upregulated DE-miRNAs suppressed the expression of these hub genes, which in turn promoted the occurrence of BRCA. Therefore, these upregulated DE-miRNAs are very likely to become new targets for BRCA diagnosis. Through the analysis of survival differences, we found that BRCA patients with high expression of hub genes LAMA2, MYH11, NR3C1, and TGFBR2 had a lower survival rate.

This high expression and low survival situation provide new opportunities for clinical treatment. Further study is required to determine the specific function. Patients with a high expression level of the FOS gene have a higher survival rate, which indicates that FOS plays an important role in the prognosis of BRCA, and related studies have also initially confirmed this important role [64, 66]. However, miR-7, which can target FOS, has been rarely studied in BRCA.

It has been reported that miR-454 is an endogenous oncogene of breast cancer, which can promote the proliferation and migration of breast cancer cells [65]. In order to further verify the reliability of the interaction network in this study through experiments, miR-454 and *PRNP* were selected for the study. The double luciferase assay verified that miR-454 could bind endogenous *PRNP*. The MTT assay results also showed that *PRNP* could inhibit the proliferation of breast cancer cells. These experimental results further increase the reliability of our interaction network and provide new ideas for the research on the occurrence and development of breast cancer.

Although we have found a large number of sample data and conducted an integrated bioinformatics analysis of the BRCA-miRNA-gene, there are still many deficiencies. We only verified the interaction of a miRNA and a gene in the interaction network through experiments, and did not verify the binding of all miRNAs and genes. We will verify the miRNA-gene interactions one by one, and confirm the results of our bioinformatics analysis through in vivo and in vitro experiments to further improve the BRCA-miRNA-gene network.

## 5. Conclusions

Our research has constructed the most comprehensive breast cancer miRNA-gene interaction network through the integrated analysis of multiple breast cancer databases, and verified the interaction of miR-454 and *PRNP* and the function of *PRNP* in breast cancer proliferation. This provides a comprehensive reference and ideas for the research on the occurrence and development of breast cancer.

## Figures and Tables

**Table 1:** Oligonucleotide sequences in the present study.

Name	Sequences (5′–3′)
miR-454 mimics	UAGUGCAAUAUUGCUUAUAGGGU
	CCUAUAAGCAAUAUUGCACUAUU
miR-454 inhibitors	ACCCUAUAAGCAAUAUUGCACUA
Mimic NC	UUCUUCGAACGUGUCACGUTT
Inhibitor NC	CAGUACUUUUGUGUAGUACAA
PRNP siRNA	GACCGUUACUAUCGUGAAATT
	UUUCACGAUAGUAACGGUCTT
siRNA NC	UUCUUCGAACGUGUCACGUTT

## Data Availability

The data used to support the study findings are mostly included in the article. It is available from the corresponding authors upon request.

## Supplementary Materials

Supplementary Materials Table S3: DE-genes and miRNet gene intersection list.
