# Supplementary material for: Construction of a Potential Breast Cancer-Related miRNA-mRNA Regulatory Network
Source: Biomed Res Int. 2020 Nov 4;2020:6149174. doi: 10.1155/2020/6149174 (PMC7657683; doi:10.1155/2020/6149174)
Supplement: Supplementary Materials — Table S3: DE-genes and miRNet gene intersection list. [file 6149174.f1.pdf]

GSE139038	GSE109169	miRNet predicted genes （down miRNA）	Intersection
UBE2T	INHBA	ABL2	BUB1
PTTG1	OLR1	ACTC1	GJB2
BUB1B	SLC50A1	SLC25A6	SPP1
UHRF1	SULF1	BCL2	CENPF
CDCA5	MMP11	DDX6	TTK
C20orf129	TPX2	FOS	MMP11
CEP55	NACC1	NR5A2	MELK
PBK	PAFAH1B3	HMGB2	MMP13
FLJ20105	DTL	HNRNPF	CTHRC1
DLG7	MRPS34	HRAS	E2F8
FLVCR1	GINS1	HSP90AA1	MMP1
ORC6L	NUSAP1	IGF1R	OLR1
KIF15	FN1	JUN	LMNB1
SHCBP1	CCNE2	KCND3	SQLE
BUB1	INTS7	KPNA2	RAD51
CCNA2	UBE2T	TNPO1	RACGAP1
DTL	CENPI	MCL1	COL10A1
TOP2A	CCN4	MET	HELLS
HN1	BUB1B	MMP11	STAT1
GJB2	EZH2	NFKB1	CST1
STK6	DPP3	NOTCH1	CXCL9
KIF11	KIF11	PDE4D	OAS2
DKFZp762E1312	TUFT1	PIK3CA	
CDCA8	ANLN	PTGFRN	
HIST1H2BL	KNL1	RAP1B	
CDC2	CDK5	RPS23	
SPP1	BUB1	RPS26	
CENPF	CKS2	RREB1	
TTK	PCNA	SMARCA4	
HES6	CENPF	SRPK1	
TUBA6	GJB2	SYT5	
KIF4A	JPT1	TAF13	
FAM54A	PRC1	TCF12	
MMP11	CDK1	KLF10	
MLF1IP	DYNLL1	TPD52	
KNTC2	TOP2A	WNT1	
CDC20	MPHOSPH9	BTG2	
FLVCR	MKI67	CXCR4	
BRRN1	WDR76	SGPL1	
CDC45L	ZWINT	GLP2R	
LOC146909	PCLAF	ROCK2	
COMP	SYAP1	PDE4DIP	
NEK2	CACNB3	USP6NL	
CENPE	ECT2	ARHGAP32	
NUSAP1	UHRF1	MLEC	
KIF20A	TUBA1C	PSME3	
APOBEC3B	SPC25	STAMBP	

KIF18A	PLEK2	SUGT1
MELK	CKAP2L	GABARAP
C1orf155	BGN	OIP5
KIAA1199	DLGAP5	ZHX2
EPR1	MCM4	CCT5
KIAA0101	COL10A1	FNBP4
C6orf129	FAM111B	SLC39A6
SLC16A3	CENPK	CLIC4
EST_AI123717	RMI1	FAM162A
CCNB1	PKM	TNRC6A
LAMP3	PLK1	TMED7
ASPM	H2BC5	HOOK1
H2AFX	HMMR	PEX5L
TK1	CEP55	YTHDF1
PPAPDC1A	COL11A1	TMEM19
CCNB2	CST1	RHOT1
MCM10	TK1	CAND1
CDC6	CIT	CTTNBP2NL
LRRC15	GPRC5A	KCNQ5
RUSC1	MAGED1	C21orf59
MAD2L1	ASF1B	DUSP22
C22orf18	PSMC4	CIAPIN1
CXCL11	HSD17B6	MKL2
KIAA1794	CHEK1	ZBTB26
MMP13	IQGAP3	RAP2C
CDKN2A	SLC16A3	ADGRL4
FN1	KIF20A	IPPK
NFE2L3	RFC4	TAF1D
BRCA2	MORF4L2	DERL1
CTHRC1	ASPM	PAGR1
KIAA1524	SPAG5	KLHL36
ESCO2	H2BC7	TMC7
ECT2	KNTC1	FBXL18
KIF14	CSE1L	CHD9
ATAD2	KIF23	SPRY4
E2F8	BUB3	LCOR
C11orf82	TSTA3	TGS1
MIAT	NUF2	PDZD8
HIST2H4B	E2F8	UHMK1
MMP1	PIP4K2C	DCBLD2
KPNA2	CANT1	ZNF480
INDO	TLCD1	B3GALNT2
CDKN3	APOO	FAM84B
HCAP-G	SGO1	PAPD4
CXCL10	CCNB2	ADAMTS17
OIP5	RACGAP1	ZNF431
SPBC24	RAD51	ZNF367
ZNF695	GGCT	SPATA13

BAPX1	GINS2	EPGN
TMEM132A	SKA3	KSR2
PTTG3	DSN1	TMPRSS12
DNA2L	RAD21	DCAF4L1
HSH2D	NCAPH	NANOGNB
HIST1H2AM	AP1M2	NCR3LG1
PLK4	LRRC15	ZBTB34
HIST1H2BK	SYNGR2	BCL2L15
OLR1	KRT19	PGAM4
SGOL2	WDHD1	ARF6
GALNT5	MELK	ARHGAP5
CHML	BRIP1	CD40
SLD5	CCNB1	CDK4
TPX2	H3C10	DOCK3
IL18	CDC6	FBN1
HSPE1	EXO1	FOXO1
HMMR	FANCI	FPR1
GPRC5A	CENPU	H3F3B
EME1	SMC4	HMGA1
RIBC2	PAQR4	HPGD
C12orf48	CYRIB	IGSF3
ESM1	SQLE	ID4
LMNB1	NEK2	LTBP2
STIL	CDC20	SMAD2
C15orf42	PRELID3B	SERPINE1
SQLE	NDC80	PCCA
RAD51	KIF4A	PIM1
PGK1	CENPE	PIK3R1
HIST1H1C	PBK	PTEN
G1P2	CTHRC1	SNAI1
PSRC1	AURKA	SP4
GPR19	RAB31	HAT1
C20orf39	FAM83D	NRP2
NUP210	H3C4	MBD4
RACGAP1	NEIL3	CLDN10
MTBP	RAB25	CRIPT
SDS	IDH2	BAG2
SAMSN1	GPR141	G3BP2
FAM64A	VANGL1	ABCF2
PRC1	LAMP5	BASP1
BLM	CDKN3	OLFM4
FAM132B	SLC9A3R1	ZNF460
S100P	FOXM1	TMED1
PAQR4	CCNA2	CIT
CD72	CD80	SEC23IP
TTF2	KIF15	SEL1L3
FLJ40629	PRR11	CADM1
TRIM25	YWHAZ	FOXP1

MGC40489	SLC35B1	UBE2S
C13orf3	RGS1	DCTN4
C6orf167	STIL	FAM46A
KIFC1	BLM	RBM22
COL10A1	DEPDC1	RCOR3
KIF23	MIF	CENPN
ANLN	PARPBP	YAE1D1
PKD2L1	CKAP2	FAM217B
LOC92312	MUC1	CCDC14
C20orf59	XRCC2	TM4SF20
WDHD1	NME1	SPRTN
IL8RBP	HELLS	CMSS1
FAM111B	NDUFAF6	UTP4
CDC25A	KIF18A	UBASH3B
PAK1	H3C2	ZIC5
HIST1H2BG	ARHGAP11A	SAPCD2
CKS2	MCM10	BTF3L4
BIC	STARD10	TTC8
ncRNA_BIC	MFAP2	MACROD2
TRIP13	PTTG1	METTL27
G1P3	MICAL2	DENND5B
IGHG1	PPDPF	ZNRF2
MCOLN2	CTPS1	ZDHHC20
PILRA	TRIP13	RBM12B
MTHFD2	RAD51AP1	ZNF286B
HELLS	FKBP4	ADD1
MAGEA6	DIAPH3	ADORA2B
TAP1	DDIAS	AP1G1
PITX1	CXCR4	AK2
CENPO	ESRP1	ALDOA
MAGEA12	PSMB5	ARNTL
AIM2	CLDN7	ZFHX3
SH2D2A	RAI14	CCND1
MGC11266	SYCP2	RUNX1
CARD15	TPD52	CRY2
RPL12	POLE2	CSRP2
TNFSF4	CDC45	CTNND1
GAJ	KIF2C	CYP1B1
MFSD2	CXCL11	DDT
RAMP1	KIF14	DR1
DEPDC1B	NCAPG	SLC26A2
GBP1	CST2	DUSP9
IFI30	SERINC2	ECE1
CHEK1	CEMIP	EN2
NMU	CXCL10	ERCC1
K-ALPHA-1	SLC44A4	FHL2
TUBB3	RRM2	MKNK2
STAT1	ABRACL	HMGN1

DIAPH3	KDELR3	HOXA10
CEACAM6	LMNB1	HOXC11
B3GALNT2	TICRR	HOXD11
G6PC	MYBL2	IFNAR2
MEX3A	IFI30	LFNG
CST1	H3C12	LIFR
SMC4L1	CLEC5A	SMAD4
DDIT4	ANKRD22	MAP1B
PRAMEF5	S100A14	MITF
CXCL9	RTKN2	MKLN1
ISL2	CISD1	NRAS
COL8A1	FCGR1A	OLR1
TRPM2	H2BC14	PKNOX1
SERPINE1	H2AC17	PLAGL2
UBE2S	H2AC16	PPIC
WDR5	FCGR3A	PRELP
CNTNAP2	H2AZ1	MAPK1
FBN2	MMP13	RBBP5
OR5M8	GAS2L3	RPL41
SLC2A6	PRSS8	SLC7A2
OAZ3	CYB561	SLC16A1
C8B	HID1	SNCG
E2F1	MMP1	CAPN15
ZIC1	TTK	SPIB
COL1A2	TDO2	TMBIM6
OAS2	RGS4	NR2F2
TMEM45A	ATAD2	TGFBR3
LAIR2	PLAUR	TSPYL1
HIST2H2AA4	FAM86C1P	UBE2D1
	TYMS	UGCG
	PNP	WNT9B
	LYPLA1	ZFP36
	C15orf48	ZNF148
	ZNF681	ZNF175
	DHCR24	PDHX
	COX6C	FOSL1
	H2BC3	AP3B2
	GRHL2	HIST2H2AA3
	MSR1	DYRK3
	PMAIP1	KHSRP
	CRABP2	KCNK5
	CERS6	HRK
	BST2	CCNK
	FXYD3	BUB3
	NKAIN1	NOLC1
	TSPAN13	RGS6
	SPINT2	SLC25A44
	STMN1	DAZAP2

CENPP	KLHL21
H1-5	NR1D2
RUNX1-IT1	MAMLD1
FGD6	PPIF
H2BC8	DCAF7
BCAS1	CFDP1
ZNF587	ZBTB18
PPEF1	APPBP2
RND1	ANP32B
SLC4A8	TGOLN2
H2BC12	CELF1
STAT1	UGT2B11
S100A16	GIPC1
OAS3	BTG3
MAGED2	PDIA5
CXCL9	SNRNP27
SLC7A5	NUDT21
MAL2	CHEK2
CLDN3	COPZ1
ZNF93	CEP162
CACNG4	ICK
DHCR7	ADGRL1
ZNF675	FNDC3A
ADAMDEC1	FOXJ3
TMEM45A	NCBP2
ANXA9	SETD1B
GATA3	ERC1
LBHD1	PEG10
TMEM97	TNRC6B
LRRC37A3	GSE1
TAP1	ARL6IP1
THY1	ANKS1A
TTC39A	FBXO46
GALNT6	TARDBP
OAS2	PISD
OR2B6	PNKD
CRIP1	NECAP1
PLAU	UBXN7
CFB	SERBP1
SLC16A6	ZBTB20
TPMT	PLA2G2D
PARD6B	TRUB2
CLDN4	NPTN
TSPAN1	TOR2A
FTL	CD274
PRR15L	USP25
GPR160	NRBP1
UGCG	ITSN2

SUSD3	NAA20
SPP1	PAIP2
PLEKHF2	SUCO
COL1A1	RAB14
PRLR	DYNC2LI1
ERBB3	NLK
REPS2	GNG13
LEF1	OTUD4
CREB3L1	PSPC1
H2BC21	NLRP2
ERP27	EIF5A2
GGH	C8orf4
GREB1L	NUFIP2
SLAMF8	GPAM
H3C3	IGDCC4
H4C8	ABHD17C
FPR3	MRPS14
MXRA5	NECAB3
FOXD4L3	ZNF106
RAB3IP	C16orf58
RET	ELOVL1
POSTN	GNPNAT1
DNAJC12	USP46
IL20	WDR77
FAP	EFHD2
OAS1	HMGN5
FOXA1	AKIRIN1
FADS2	MANEA
SEPTIN3	ALG9
MRPS30	METTL8
NAT1	SEMA6D
MMP12	TMEM254
SLC27A2	WDCP
IFI6	COL18A1
EPYC	TXNDC5
MS4A14	TOMM40L
SPDEF	GTF2IRD2
ICOS	YIPF4
GSDMC	PIGO
EPCAM	MIGA2
DIO1	FAM136A
CHST1	MED30
C4B	ANKRD40
COL12A1	UBXN11
NPNT	LONRF1
ZNF552	SFXN1
GBP5	VPS26B
ENPP5	SLC25A25

GREM1	OSBPL10
IDO1	ZNF618
CEACAM5	KCTD12
H3C11	KLHDC3
KMO	SFXN4
AGR2	ISCA2
BAMBI	LIN54
BPIFB1	TMCO5A
AGR3	TIGD2
CASP14	MIER3
SYT13	ZNF800
ZG16B	ZNF384
CEACAM6	RNF152
MYB	ARL5B
IFNL2	ZBTB38
CYP2B6	HS6ST3
CA12	PROX2
ASPN	RGPD4
BMPR1B	C5orf51
ABCA12	KRTAP13-2
CD24	GPIHBP1
TFF1	MACC1
CLEC3A	SLC6A17
CYP2B7P	BDNF-AS
PVALB	TMEM91
GRPR	HIST2H2AA4
	NDUFC2-KCTD14
	ATP6AP1
	CREBBP
	CLN8
	PLXNA2
	TROVE2
	YWHAZ
	NFS1
	PKMYT1
	KCNK6
	KLHL3
	TMED9
	GRAMD1B
	SINHCAF
	CCDC90B
	ZNF585B
	ZFPM1
	MCOLN2
	POTEG
	POTEM
	GRK3
	ALCAM

RHOA
ART4
ATP5G1
CAPZB
CBS
CKB
FOXC1
GFRA1
GUCA1B
IFNAR1
IPP
MLLT1
MLLT6
MUC3A
NDUFV3
NFIX
NOTCH3
NUCB1
P4HB
PRKN
PDGFRA
PGAM1
MAPK3
PSPH
PTMA
PTPN14
PURB
MAP4K2
SMARCD1
SRF
TRAF1
VHL
XPC
ZNF708
ZNF70
API5
SYT7
RASSF9
MAPKAPK2
HAND2
AKAP6
IGDCC3
CCS
GNE
GPC6
G3BP1
PLIN3
STX6

EMC8
SPON2
LYPLA1
GMEB1
SRSF10
POLR3A
RHOBTB3
MLXIP
HIC2
TNFAIP8
BACE2
HEATR5A
KANK2
HINFP
NTMT1
ORMDL2
TIMM22
IL21R
TRAT1
WDPCP
PHF20
ETV7
LARS
GDE1
DPP8
THAP1
MIOX
PEX26
IPO9
MINDY1
FOXJ2
UTP6
LMOD3
RNF20
BARHL1
MAVS
TAOK1
KIAA1456
KIAA1549
CACNG8
CLSPN
FN3K
SUSD1
FAM160B2
PLEKHG2
ZNF747
PPDPF
ZYG11B

ZMYM1
ZNF556
FAHD1
KREMEN1
C9orf64
MCRIP2
RRP36
NAV1
KIR3DX1
TIMM29
ZNF276
ZFAND4
ZNF101
PRRT2
FLYWCH2
ABHD15
BORCS7
SPPL3
ZNF813
C19orf47
RNF19B
LAYN
ALG10B
GPR156
APOBEC3F
DENND6A
RSBN1L
PCSK9
NEK8
FAM83H
ILDR1
ZNF677
SLC35B2
ZNF662
ZNF793
ZYG11A
ARGFX
GDF5OS
ADCY9
AP2B1
AKT1
APEX1
ATP1A1
ATP5E
CAPNS1
COL4A1
DDX3X
DHCR24

DLD
E4F1
EIF5A
MARK2
EXTL3
FGFR3
FKBP5
FOSL2
MTOR
GLUD1
GNAL
GOLGA3
GTF2H1
GTF2I
HADHB
HOXA1
IFIT3
MEF2D
COX2
ND3
MYL2
NDUFA2
NFE2L1
ORC5
P2RY11
PLSCR1
PPM1A
PPP1CB
MAPK6
PSMA2
RAD51C
RARS
RB1
RCN2
RPL37A
RPL36A
RPS6
RPS15
SALL2
SEC13
SKI
TUBG1
TYMS
UBB
CCDC6
CUL3
CUL2
SMARCA5

DDX18
MTMR3
DNAJA3
ITM2B
RASAL2
PUM1
DDX46
MED12
UBA2
CTDSPL
TRIB1
NPM3
CERS1
LMAN2
AKAP2
VPS45
DDX42
MON1B
SNRNP200
AGO1
AGO2
COQ2
NFU1
SMPDL3B
TRIB2
N6AMT1
BRD7
UQCR10
GRHL1
NOX4
MRPS33
TPPP3
NDE1
TTC38
RAVER2
SYNJ2BP
N4BP2
TMEM30A
CBWD1
JPH1
SPIRE1
TOMM22
ADGRG6
POGK
ZBTB4
SUDS3
STEAP4
FOXRED2

CTC1
DDHD1
MCM8
KBTBD8
ADO
TMEM209
ORMDL1
LRIG3
WHAMM
METTL23
SGPP2
DOCK11
RNF187
RUNDC3B
FAM171B
GXYLT1
RPL7L1
GTF2H2C
ABCF1
ACP1
AR
BCL6
RUNX2
TNFSF8
CDK1
CDK6
CENPF
CREB1
MAPK14
CTGF
DDX5
E2F1
E2F5
PHC2
ERBB2
ERBB3
ESRRG
ETF1
F2RL2
ACSL1
ACSL4
GM2A
GNAS
GOT1
HMGB1
HMGB3
HOXA11
DNAJA1

HSPA8
CYR61
INPPL1
ITGA5
KCNJ10
LAMC1
LMNA
LRP1
LYN
MARCKS
SMAD1
MAOA
MDH2
MAP3K9
AFF1
NDUFA4
NDUFB2
NOTCH2
NSF
MED1
PRKCE
PRLR
PTPRJ
PTPRM
RAN
RAP2B
RBBP4
CLIP1
VPS52
SQLE
SRC
SRD5A1
VAMP1
TAF11
ZEB1
TCF20
PRDX2
TMSB4X
TP73
UVRAG
VEGFA
EZR
YES1
YY1
YWHAH
CUL5
NPRL3
PICALM

XPR1
IL32
B4GALT6
B4GALT5
TXNL1
TM9SF2
SERTAD2
ZEB2
MED13
ANGPTL7
SIGMAR1
SMNDC1
RTN3
IPO7
NFAT5
SERINC3
IL24
XPOT
MGLL
CPEB3
ENPP4
STK38L
PHLPP2
PHF8
DMXL2
SATB2
SLC39A14
MMD
BAMBI
TRAF3IP1
AFF4
KLHL5
SARAF
PANK1
TOLLIP
CCNJ
SPDL1
IMPAD1
C1orf123
LRRC59
ETNK1
YEATS2
BDP1
KCTD16
ZSWIM5
PHF12
NCAPG
RFX7

MRPL44
UBE2Z
PRRG4
NOX5
LPCAT1
LRRTM4
RAB11FIP1
SLC38A1
NIPA2
GUCD1
TEX35
MAML2
ACTRT3
PARD6B
PIP4P1
CDKN2AIPNL
NEK9
EGLN2
TMEM123
LYSMD3
BORCS5
C11orf74
LRRK2
EID2B
VTI1A
RNF217
AMOT
SLC5A12
TMEM201
JMJD1C
SMIM13
PRR15
KCTD20
SLC41A1
CFAP65
PLCXD2
BCL9L
SHISA6
PAIP2B
C6orf201
PAPPA-AS1
TMEM239
ABAT
ADH5
JAG1
AKT2
AIRE
XIAP

BRAF
KLF5
CALM3
CASP5
CD44
CDC25B
CCR6
COL1A1
COL3A1
COL5A1
COL5A2
COX6B1
CYP2C9
DBT
TIMM8A
DNMT3A
DTNB
EPB42
FHIT
GLUL
HK2
HNF4A
SP110
IGFBP5
IL10RB
IL13RA1
ITGB1
ITGB4
KCNJ3
KRAS
LIMK1
SMAD3
MDM2
MMP2
MMP9
MMP13
MMP14
MYO6
NFATC1
NFIC
NFKB2
NFYB
NOS1
NPR1
OAS3
OAZ1
PAPPA
PDGFB

PPP2R2A
PPP2R5E
MAPK7
KLK10
PTGS2
BRD2
RPS19
RPS27
SCN2B
SDC1
SMARCD2
FSCN1
SNX2
STAR
MAP3K7
TERT
TFPI
TLR2
TNF
NR2C2
TUBB2A
TULP3
UBE2V2
UCK2
YWHAB
GLRA3
SLC25A16
HIST1H2BG
YARS
AKR7A2
SKAP2
ATP6V0E1
LRAT
ZNF264
CHST10
ADAMTS4
SPTLC2
BAG3
SECISBP2L
JAKMIP2
SLC30A9
C1orf61
DCTN6
NCKAP1
LILRB1
RER1
KAT7
NUDT3

ZNF277
GALNT6
IKZF3
GGA2
METAP1
RCOR1
KIAA0930
HAAO
ACOT9
GABARAPL1
HYPK
PGLS
TECPR1
MYCBP
OSTF1
BBC3
RRP7A
ZBTB44
DNTTIP2
COMMD2
IER5
CMPK1
PHAX
RBM27
TMEM214
RNF125
SLC35F6
TMEM33
PNPO
ELP2
ELMOD1
FGD6
STOX2
GJD2
RAB22A
MTA3
PLEKHA1
FNDC3B
C1orf50
ZNF426
GLB1L
LRRC2
CERS4
TRMT2B
CCDC170
NUBPL
ORAI2
TTYH3

APOL6
VOPP1
CSRNP2
RAB33B
MAGT1
USP42
SLC25A33
HOOK3
FAXC
ZBTB37
MYPN
PRRC2B
ZNF607
LMNB2
PPIL4
ZC3HAV1L
PLCD3
FAM83F
OSBPL8
AGAP1
ANKRD9
FSD2
ZNF440
SWSAP1
ZNF573
TRIM71
FAM241A
SAMD8
SLC2A14
RUNDC1
C2orf15
FAM120AOS
OR7D2
CREG2
NAPEPLD
ANKS4B
LRIT3
FAM71F2
ZNF429
SFT2D2
ENO4
CLEC17A
PLEKHM3
FAM102A
CENPP
CTXN1
GTF2H5
PRR23A

ZNF878
ACTB
ADD3
CRYBG1
ALDH3A1
ALPPL2
ANGPT2
BNIP3
BTG1
VPS51
MYRF
CBFB
CD28
CDH2
CDKN1A
CFTR
CYP2C19
DDC
DMXL1
DFFA
HBEGF
DUSP6
E2F3
EGFR
EIF4E
EPAS1
ERBB4
ERG
ESR1
ETS1
FLI1
FXN
GMFB
HDAC2
HIF1A
HOXA9
IFNB1
ILK
IRS1
ITGB8
KRT7
SMAD5
MCM2
MAP3K3
MEST
MAP3K11
MMP1
MMP12

ABCC1
MSH3
NUDT1
MUC1
MUC4
MYC
MYO5A
NAIP
NDUFS2
NEDD9
ORC4
P4HA1
PIGF
PODXL
POU5F1
PPP3CA
MAP2K6
PXN
REL
ROBO2
ROCK1
RPA1
RPS6KA3
RPS6KB1
RTKN
MAP2K4
SET
HLTF
SNTB1
SOX2
SOX9
SOX11
SP1
SPTBN1
STAT1
ADAM17
TGFB2
TGFBI
TGFBR2
TSPAN6
TNR
TPM3
ZBTB25
PRDM2
DEK
PTP4A2
HMGA2
FZD6

FZD7
HIST1H2BF
PPM1D
NIPSNAP1
AGPS
IRS2
TNFSF13
SLC16A5
KLF4
ZFYVE9
CLINT1
VGLL4
AQR
PHACTR2
PLEKHM1
PAK4
FAM3C
DDX17
ARL6IP5
SPTLC1
IVNS1ABP
LYPLA2
SWAP70
ANKRD28
ZNF451
WSB1
SRPX2
SNX24
TMOD3
SENP1
PADI1
PNMA3
PSAT1
SOCS7
F11R
APH1A
GOLM1
DNAJC28
C1orf27
TUG1
UBR7
KIF21A
TMEM9B
CTNNBIP1
AKR1B10
AGTRAP
CAMK1D
NDRG2

SRGAP1
CRAMP1
ABRACL
ZFAND3
CLSTN2
RRAGC
MTMR14
PARP8
HDAC11
NANOG
JADE1
CSRNP3
PDGFD
CPEB4
MAP1LC3B
SESN2
MIXL1
IMMP2L
UTP15
DDI2
CEP19
HIST1H2AH
MTDH
RBM18
DTD1
MYOCD
SLC22A9
TIRAP
RAB3IP
SLC16A10
SP7
CCDC43
UNC5D
SMIM17
CCDC80
C11orf65
ZNF100
UBXN2A
AAED1
THSD7A
WASHC2C
BCLAF3
SERINC5
MUC19
ZNF660
TPRG1
CCDC85C
ZNF678

NUP43
ZNF445
SAMD5
ZNF772
FAM45A
CRNDE
AVPR1A
COL19A1
CRK
CSNK1G3
CYLD
DSG2
DSPP
DVL3
ELK4
ERCC6
FOXO3
FYB1
GABRA4
GATM
GRIK5
NR3C1
MR1
HOXB6
HOXC8
MRPL58
IFIT1
IL6R
MMP7
MYBL1
MYCN
NPTX1
PGM3
PLAG1
MAPK8
PTPN7
CXCL12
SMARCC2
SON
TFRC
UGT8
ZNF180
ZNF223
ZXDA
NCOA3
SERF1A
SEMA7A
AOC3

GALNT4
ADAM9
NAPG
MBD2
SOCS3
VAPA
SRSF11
SCAMP1
BZW1
HNRNPDL
SLC12A6
SMG1
MYCBP2
KIF1B
CBX5
POFUT1
CHMP2B
SNX10
ERGIC2
CRNKL1
ZBTB7A
BTBD1
LEPROT
ARGLU1
SETD5
TMEM100
CSGALNACT1
SLAIN2
GPBP1
WNK1
C8orf33
LONRF3
L2HGDH
DCAF17
ZNF703
NAA50
COQ10B
COASY
TRIM8
ARID5B
ZNF566
SLC45A3
TMEM182
SPIN4
SPTY2D1
C18orf25
SPRED1
AGO3

VMA21
BRWD3
ZNF326
ZBTB41
ZFP62
SERF1B
HOTAIR
POC1B-GALNT4
ACVR2B
ADM
ADGRE5
CRKL
DNMT1
EZH2
GRIN2B
IGFBP2
KCNJ1
LRP6
NFKBIA
PGR
PIK3CG
PIK3R2
TWF1
RGS3
TCF4
TEK
VCAM1
EVI5
SLC7A5
CCNE2
TOM1
MERTK
PLK2
TWF2
SIRT1
SZRD1
PITPNC1
RBMX
EGFL7
RHOU
Cdkn1b
SLC41A2
TMEM50B
CCND2
GATA6
LMNB1
MYO9B
PPP1CC

RAB5B
RPS4X
AURKA
WNT5A
FZD9
PER3
TAF1B
UST
ZNF267
ATG7
MYL12A
CBX3
ZFP30
DICER1
ACAP2
VSIG2
ULK3
SESN1
NRBF2
SAR1B
WAC
GALNT7
SNRK
ATG2B
SLC25A36
C11orf57
ASH1L
CLDND1
OTUD7B
KIAA1191
ANKEF1
PLEKHF2
PCNX2
SLC25A28
DNAL1
TMEM79
LZIC
PCGF5
FAM126A
ZXDB
TET3
MMS22L
PLA2G4D
MTRNR2L9
KPNA3
MMP16
PRKAA1
RORA

SYPL1
TADA2A
BRD3
CHAF1B
TRIM66
ACTR2
PLEKHA6
NGDN
ZDHHC2
FOCAD
FIGN
PLXNA3
TDRD1
AKIP1
PMEPA1
TULP4
GID4
PANK3
MCMBP
MRI1
HPS3
LIN52
CLEC12A
KANSL1
ATP11C
AQP3
ARL2
DST
CACNA1A
CANX
CD3D
CDC27
MAP3K8
CPT1A
CPT1B
CTNNB1
DCK
GADD45A
FOXM1
AFF2
HNRNPA1
HSP90AB1
IFNGR2
ITPA
KCNN1
KLK2
MGMT
NF1

NFIB
PEBP1
MAP2K2
SCN7A
ZNF236
RAB7A
SLBP
LZTR1
FOXH1
TCEAL1
BAG4
KIAA0355
ARID3B
SRCAP
BLCAP
CKAP4
MAPRE2
NFASC
RAD54L2
NUP210
PTCD1
MTG2
ZNF330
PARVB
DSE
MRM2
EHD3
IRAK4
SPA17
MYO3A
HMGCLL1
SPATA6
HES2
LY6K
RETSAT
LAX1
DHX33
VN1R1
SFMBT2
SEMA4G
NUCKS1
KCTD15
LIN28A
TRAF7
C9orf3
PRPF38A
EAF1
PNPT1

LRSAM1
PYGO2
ATP6AP1L
CENPBD1
GPR146
RAB3C
SENP8
GALM
ZBTB46
HNRNPA1L2
EXOC8
VKORC1L1
LINGO2
KIAA1958
FADS6
CYB561D1
SEC14L4
ZSWIM9
PEAR1
NSUN4
SPRED3
ZBTB8B
ATP5J2-PTCD1
CASP8
CDK2
COPA
FGF2
FOXG1
GNAT1
IAPP
IL7
KIF5C
MAD2L1
SMAD7
NDUFS3
PFKFB2
PPP3R1
PRKCH
RS1
SORT1
SNRPB
TTK
UBE2A
UBE2D2
CD164
IQGAP1
INA
RIMS2

YAF2
B3GALT5
CXCR6
ZNF266
HNRNPA0
RPP14
RAB21
ATG2A
PDS5A
ZNF281
CD2AP
LRRC6
TMEM245
WIPI2
RANBP6
KLF15
HCFC2
MRPL51
NIP7
AMOTL2
SRRT
SHC3
RNF216
C4orf19
ZNF253
ERO1B
MEPE
ZNF667
ZFP69B
ZNF669
TNKS2
SLC35G2
ZNRF3
DCTN5
ZNF594
ATG4C
KIAA1671
STON2
ZNF439
PIGM
MYSM1
LRRC58
CNTROB
WDR17
C1orf216
ACVR1C
C2CD4A
ZNF791

ZNF567
ZNF781
ZNF780B
ZNF169
PIKFYVE
FOXK1
ZNF844
CYP27C1
ZNF699
RAB15
BEND4
ZNF487
ZNF736
CASTOR2
ACTN4
APOH
ASTN1
ATM
C1QBP
CACNB2
SERPINH1
CHD2
CCR4
CSTF1
DHODH
S1PR3
EIF4A1
EMP2
ENSA
F2R
FEN1
FPR2
GABPA
GP2
GPI
HAS2
HFE
ONECUT1
PRMT1
HSPA1B
IDE
IMPDH2
IRF1
ITGAL
IVD
KCNMB1
KCNN3
LAIR1

LGALS8
LRPAP1
MAN2A2
CHST6
MYO1B
COX1
MTHFD1
NODAL
NPHP1
NPHS1
P2RX7
PDE6A
PIK3C2A
POLR2F
PSMC1
PSMD3
PYGB
RAD51
RANBP2
RPL18A
RPL24
RPL37
RRAD
RTN2
TRAPPC2
SIAH2
SLC1A5
SLC4A1
SLC15A2
SORD
SSTR2
TALDO1
TERF2
TPR
TTC4
UQCRB
USP1
WNT7B
ZNF37A
ZNF43
ZNF84
TRIM25
ZNF207
ZNF217
MOGS
BAG6
SSPN
GAN

DEGS1
EIF3D
VAMP4
WISP3
ALKBH1
EIF2B2
TM4SF5
SLC28A1
TRIP11
LIPG
STOML1
TMEM59
ENTPD4
CYTIP
NUP155
TESMIN
ZNF646
SART3
TCAF1
MELK
LRIG2
AREL1
SEC24D
RABGAP1L
IQSEC1
FGF19
PARP2
SCAMP2
ARPC2
RNF41
CD96
DLEU1
TLR6
STK25
PAICS
LEFTY1
AP4B1
KIF1C
PPARGC1A
LIAS
DDX19B
TUSC2
HSPA4L
AAK1
CLUAP1
ZBTB43
MAPK8IP3
ARC

UBR4
ADAT1
PHLDA3
NUP62
SGK3
DNAJB5
FBXL2
MOB4
FGFR1OP2
TIMM10
AKAP8L
RNF11
SIGLEC8
HCAR1
PDLIM3
POLM
FLVCR1
MCTS1
WDR91
TMOD2
YPEL1
GPSM2
HILPDA
SDF4
PIGP
NT5DC3
KLF13
SUFU
PTOV1
POLE3
CYCS
WDR5B
ING3
SGTB
FBLIM1
FAM46C
PIGG
CWC25
CMTM6
TRMT10C
ZSCAN2
HAUS2
MRPS10
MRM3
DCUN1D2
KLHDC8A
ASXL2
UEVLD

SLC35E3
DHTKD1
PIP4P2
ZCCHC8
HIF1AN
ENAH
WDR12
PRR11
CMTR2
PAG1
KLHL7
SAR1A
NLN
AARS2
ZFP14
TNRC6C
GATAD1
CACTIN
HRH4
CHTF18
ZMAT3
TTC31
SMURF2
MEAF6
KXD1
MFSD11
ZNF576
TTPAL
FYCO1
PPP1R3B
RTL10
UBA5
ABHD18
OPA3
SLC35F5
ZNF430
LRRC27
STARD5
ZNF34
HM13
ISG20L2
MARVELD1
EIF2A
BRIP1
PCBD2
QRFPR
SYDE2
CHCHD5

NOA1
SPIRE2
ARHGEF39
PPP1R15B
ATCAY
MICALL1
DNAJC14
GTPBP10
MYADM
MYOZ3
TIMM50
CYP2U1
LACTB
TMEM132B
WDR31
ZNF554
GBP4
WDR92
GINM1
NXPE1
NXPE2
C15orf40
CYB5D1
IRGQ
SHE
C20orf144
ICA1L
CMBL
ZNF786
MANEAL
IL23R
SH3D19
ITPRIPL2
ZNF519
DCP2
FAM9B
PAOX
PLD5
APOBEC3A
TRIM65
ZNF584
TMEM154
CYB561A3
SLC16A9
ZNF485
LCLAT1
UBN2
C2orf72

NWD1
FAM126B
CYP4V2
MMAB
FFAR4
ZNF517
OTOG
STAC2
ANKRD62
ANKRD36
ZNF321P
VMAC
LRRD1
HACD4
C10orf105
ZNF724
PALM2-AKAP2
TRIM72
ARIH2OS
CCDC30
ERVMER34-1
MTRNR2L5
HSPE1-MOB4
C8orf44-SGK3
ACACA
ALDOB
BYSL
CPT2
CSTF3
CYP19A1
DHX15
CYB5R3
DVL1
ECH1
EEF1B2
ENO1
BPTF
FLNA
GLI3
GRB2
HIST1H2BD
NDST1
ID2
KCNJ6
LAMP1
LDHA
MLF1
NME4

OCRL
PCCB
PML
PSMA3
PTBP1
REST
RPS7
TRA2B
SOD1
SREBF1
STXBP1
STXBP3
MLX
TCP1
UBA52
VIM
EIF4H
ZBTB14
ZNF239
KDM5C
HIST1H3H
DYRK2
KSR1
CPNE1
MSC
ZNHIT3
WTAP
OXSR1
HIPK3
DDX39A
MXD4
TXNIP
IGF2BP1
TOB2
TRAFD1
RNPS1
CASC3
SEC31A
CEP164
ZNF609
TTC28
KPNA6
PRKD2
ZNF473
PNISR
GMEB2
RDH11
MRPL37

LGSN
CAB39
ERRFI1
NDFIP2
VPS13C
FAM118A
ARL8B
WDR33
AGK
RCC2
CELF4
STARD7
CYP20A1
ESYT2
NLGN2
KIAA1468
RBAK
SLC22A23
DCLRE1B
DCTPP1
HAUS3
VPS37B
CPSF7
RABEP2
KLHL15
WNT10A
TSEN2
NDFIP1
SLC25A2
USMG5
GOLT1A
EDARADD
CHCHD4
PPARGC1B
KLF14
TMEM199
DPY19L3
Gzmb
TIGD1
NPNT
B4GALNT3
CHAMP1
C3orf38
ASB16-AS1
BLOC1S3
GOLGA8B
PARP1
CD59

EDN1
EPS8
HSPA5
IFRD1
IL11
LNPEP
MCC
MDM4
METTL1
YBX1
PAFAH1B2
SERPINA1
PTK2
RPL7
RRM1
SLC20A1
TGFBR1
PABPN1
ALDH4A1
PEX11B
SLC5A6
WASL
ZRANB2
MICAL2
TSC22D2
TRIM13
PCGF3
TDRKH
SF3B1
NEPRO
POLL
MRPS18B
ABT1
ABHD5
TUBD1
TRPV2
MTRF1L
TBC1D19
FAM222B
CISD1
NDUFA12
SLC2A11
C1orf115
EDEM3
TM2D2
RAB11FIP4
ZSWIM1
PRRC1

BRI3BP
TRUB1
BEST3
TOR1AIP2
IFNLR1
PDE12
NUDT8
ELMOD2
GDPD1
NHLRC3
ZNF749
LIN28B
FAM83G
ADH1B
ARL1
CD1C
CLTC
COX6A1
CSTF2
DLX2
EIF4A2
EN1
GJA1
MSH6
ID1
INSIG1
ITGA3
LDLR
MBNL1
MID1
P2RX5
PPP1R10
PRKCB
PTPRS
PURA
RAC1
RPS16
MSMO1
SLC6A8
SMARCB1
SNTB2
SP100
HSPA13
TWIST1
WEE1
ZNF136
VEZF1
LUZP1

FZD5
SHOC2
SPAG9
MYOM2
COPB2
C14orf2
HDAC4
C2CD5
ABCC4
SEMA4D
PRPF8
SEC24A
ZNF652
CNKSR2
TBC1D9
TTC33
POLA2
DSTYK
MGAT4C
SYF2
TES
GREM1
HBP1
BRPF3
HSD17B12
TRIM44
OCIAD1
CLN6
SLC35A5
ANO1
NDUFAF7
RUFY2
VAC14
SIPA1L2
CREBZF
AASDHPPT
SCOC
PAPOLG
E2F8
PUS3
CRISPLD2
SFT2D3
CASC4
SLC2A13
ARAP2
ANAPC16
SLC36A4
DCUN1D3

UBXN2B
ASB11
SLC16A14
CREBRF
ZNF585A
ATXN7L1
SGMS1
NAP1L5
COX6A1P2
LURAP1L
ZNF181
ZNF546
IRF2BP2
PRR5-ARHGAP8
MTRNR2L2
MTRNR2L8
SLFN12L
SERPINA3
ABCA3
ABCA4
ACR
ACVR1
ADAM8
ADCY6
ADCY7
ADH6
ADH7
AGRP
ALDH2
ALDOC
AMELY
AMHR2
AMPD3
BIN1
AMT
AMY1B
AMY1C
ANG
ANK2
ANXA1
ANXA11
APAF1
APBB1
APBB2
BIRC5
APLP1
APOC1
KLK3

AQP5
AREG
RHOB
ARRB1
ARSA
ARSF
ART3
ASAH1
ASCL2
ASGR1
ASIP
ATOH1
ATP1B2
ATP2B4
ATP6V0A1
ATP7A
AVP
AVPR1B
BAAT
ADGRB3
NKX3-2
BCHE
BCL2A1
BCL2L2
BCL7A
OPN1SW
CFB
BIK
BNC1
BMP1
BMP2
BRCA1
KLF9
SERPING1
C1QA
C1S
C2
C5AR1
C8A
C8G
MRPL49
MPPED2
DAGLA
LDLRAD4
CA3
CA5A
CA12
CACNA1B

DDR1
CACNB3
CACNG1
CALB2
CAMLG
CASQ1
CCNG2
CCNT1
CD1D
CD5L
CD8A
CD9
CD14
CD22
CD24P4
CD27
CD33
CD36
CD37
CD38
ENTPD3
CD69
CD79A
CDA
CDH3
CDH5
CDH11
CDKN1C
CDR1
CDS1
CDX2
CDX4
CEACAM5
CEL
CGA
CEACAM3
CEACAM8
CHAT
CHI3L2
CHRM2
LYST
CHRM3
CHRNA7
CHRNB4
CLCN2
CLCN3
CLCN6
CLIC2

CCR7
CNGB1
PLK3
COL4A5
COL6A1
COL8A2
COL9A2
COL9A3
COL10A1
COL11A1
COL15A1
COX8A
CPE
CLDN4
CLDN7
CRABP1
ATF2
CRHR2
CRIP2
HAPLN1
CRYBA1
CRYBA2
CRYGC
CRYGS
CSF1
CSN3
CST1
CST2
CST3
CST4
CST6
CTH
CTRB1
CTSD
CTSW
CYB561
CYP2E1
CYP2F1
CYP3A5
CYP7A1
CYP21A2
CYP21A1P
CYP26A1
CYP51A1
DAB2
DACH1
DAP
DAZL

DBH
ACE
DDIT3
DEFA4
DEFB4A
DHCR7
1\Sep
DIO1
DLG2
DLG3
DLX5
DMPK
DNASE1L3
DYNC1I1
DNMT3B
SLC26A3
DRD5
DSC2
RCAN1
TSC22D3
DTX1
DUSP5
DYRK1A
EBF1
EFNA1
EFNA2
EFNA5
CELSR3
MEGF9
EGR3
SERPINB1
EMP1
ADGRE1
EMX2
EPHA1
EPHA4
EPHB4
EPOR
NR2F6
EREG
ESR2
ESRRB
ETV1
ETV5
EVC
F3
F10
F13B

FABP4
FABP1
FABP6
ACSL3
PTK2B
FBLN2
FBN2
MS4A2
FCGRT
FDFT1
FGF4
FGF8
FGF10
FGF11
FHL1
VEGFD
FOXI1
FOXD2
FLT3
FOLR2
FRK
ADAM2
G6PD
GAGE1
GABBR1
GABRA1
GABRP
GAGE2C
LRRC32
GATA1
GBP2
GBX2
GC
GCH1
GCK
GCNT2
OPN1MW
GDF9
GDF10
GEM
GFAP
GFPT1
GGT5
GIP
GJA8
GJB1
GJB2
GJB5

GLG1
GLI1
GLRA2
GLS
GNA12
GNGT1
GNGT2
GNS
GOLGB1
GP9
GPD2
GPR17
GPR22
GPER1
GPR34
GPR37
FFAR1
GPX2
GRB7
GRB10
GRIA1
GRIK4
GRIN2D
GRM3
GRM4
GRM5
CXCL2
CXCL3
GSN
GUCY1A2
GUCA2B
GZMK
HBE1
SERPIND1
HCRTR1
CFHR2
HGD
HGFAC
HIC1
HLA-DMA
HLA-DRB5
HMGCR
HMGCS1
HMOX1
NR4A1
FOXA2
FOXA3
HOXA2

HOXB1
HOXB3
HOXC6
HOXD3
AGFG2
HRC
HRH2
HSD3B2
HSD11B1
HSD17B3
HSPA1A
HSPA1L
HSPG2
HTN3
HTR1A
HTR1D
HTR7
TNC
ICAM1
IDH1
IDUA
CFI
IFI16
IFNA21
IGFBP4
IGLC1
IGLC2
IGLC3
IKBKB
IL1A
IL4
IL5
IL6
IL6ST
CXCL8
CXCR1
CXCR2
TNFRSF9
IL17A
INHBB
IRAK2
IRF2
IRF6
ISG20
ITGA1
ITGA2
ITGB2
ITGB5

ITGB6
ITIH1
ITPR1
ITPR2
IVL
JAG2
JAK2
JAK3
JUND
JUP
KCNA4
KCNC1
KCNH1
KCNH2
KCNJ9
KDR
KIT
KLRB1
KLRC1
KLRC2
KLRC3
KPNA5
KRT4
KRT5
KRT19
KRT32
L1CAM
LAIR2
LAMA5
LAMB1
LBP
LCK
LCN1
LEPR
LGALS9
LIF
LIM2
ABLIM1
LIPA
FADS1
LLGL2
LOX
LOXL1
LRP5
LSAMP
LSP1
LSS
LTBP1

LTC4S
LTK
LY6H
LYL1
TACSTD2
MXD1
MAGEB1
MAN1A1
MANBA
MAOB
MAP2
MAT1A
MATK
CD46
MDFI
DNAJB9
MEF2A
MEOX2
MFGE8
MFNG
MGAT3
SCGB2A2
MGP
CIITA
CXCL9
MLN
KMT2A
AFDN
FOXO4
NR3C2
MMP17
MN1
MUC5AC
MUC6
MXI1
MYBPH
MYCL
MYO7A
MYOD1
NAB1
NAB2
NCAM2
NCF4
NDP
NEFM
NEFH
NEUROD2
NEUROG1

NFIL3
NGFR
NKG7
NKTR
NOS3
NPY
NOVA1
NPAS1
NPAS2
NPC1
NPR3
NPY1R
NTF3
NTRK2
NTN3
ROR2
NTSR1
NUMA1
NR4A2
OAS1
OAS2
OGDH
SIX6
OR3A1
OR3A2
SLC22A18
OSBP
OVGP1
P2RX1
P2RX4
P2RY2
PAH
PAK3
PC
PCDHGC3
CDK17
CDK18
PDE2A
PDE4A
PDE6G
PDE6H
PDK4
SERPINF1
PEG3
PER1
CFP
PFKFB4
PGM5

SERPINB5
SERPINB9
SERPINB10
PIK3C2B
PLAT
PLAUR
PLCD1
PLCG2
PLTP
PLXNA1
PNLIP
PNLIPRP2
POMC
PON3
POR
POU6F1
PPARA
PPBP
PPL
PPP1R1A
PPP1R3C
PPP1R3D
PPP2R5A
PPP3R2
NPY4R
PRH2
PRKCG
PRKG2
MAPK4
MAP2K5
DNAJC3
PSPN
PROS1
PROX1
PRRG2
KLK7
PRSS8
PSAP
PTAFR
PTGDS
PTH
PTPRA
PTPRE
PTPRH
PTPRN2
NECTIN2
PCYT2
PYGM

RGL2
RAB6A
RALGDS
RARG
RARRES2
RASA1
RASA2
ARID4A
KDM5A
RBL2
RBMY1HP
RBP1
OPN1LW
RCVRN
RDH5
REN
RFX2
RFX3
RGS13
RGS16
RNASE2
RNASE4
RNASEL
RNU4-8P
RORB
RORC
RP1
RPE65
RPL12
RPS6KA2
RRBP1
RXRB
RYR2
S100A5
SAG
SAT1
SATB1
SC5D
ATXN1
SCD
CLEC11A
SCN1B
SCN3A
SCN4A
SCN9A
SCNN1D
SRL
CCL14

CCL15
CCL19
CCL20
CCL24
CCL25
SEL1L
SELPLG
SFRP1
SFTPD
SGCD
SHH
ST6GAL1
PMEL
SLC1A1
SLC1A4
SLC2A3
SLC2A4
SLC5A1
SLC5A3
SLC5A4
SLC6A4
SLC6A6
SLC6A9
SLC6A12
SLC12A3
SLC17A1
SLC25A1
SLC22A2
SLPI
SNCB
SOS2
SOX1
SOX4
SOX5
SPP1
SPRR2G
SPRR3
SPTB
SPTBN2
SREBF2
ST14
STAT2
STAT6
STX1A
STX3
SULT1C2
SULT2A1
ABCC8

SVIL
SYN1
SYP
SYT1
TACR2
TACR3
TAF1
TAZ
TBL1X
TBX5
TBX15
TBXAS1
TCEA3
TRAV6
TECTB
TF
TFAP2B
TFAP4
TFE3
TFF2
THBD
THBS3
TIAM1
TIMP3
TK2
TLE4
TLL1
TLR1
TLR4
TM7SF2
CLEC3B
TNFAIP2
TNNC1
TNNI1
TNNT2
TNS1
TPD52L1
TPO
HSP90B1
TRPC1
TSC1
TSHR
TTC3
UBE2H
UGDH
UGT2B4
UGT2B17
USF2

USH2A
VIP
VLDLR
VTN
CLIP2
WNT3
WNT10B
WNT9A
ZNF711
ZNF35
ZNF76
ZNF221
ZNF135
ZNF157
ZKSCAN8
SLC30A2
DNALI1
LAPTM5
NPHS2
SEMA3B
ST8SIA4
PLA2G7
FGF23
GDF5
TKTL1
KDM5D
COLQ
HIST1H4I
TRRAP
KLRC4
AXIN1
FZD1
FZD8
HIST1H3C
HIST1H4L
PLA2G10
ULK1
EEA1
BFSP2
LTBP4
RECK
SOAT2
TEAD2
SORBS2
CDC42BPA
PPFIBP1
PPFIA4
PPFIA2

ITGA10
IFITM1
CBX4
BARX2
MAPKAPK5
BHLHE40
PDLIM4
KLF7
PLPP3
USO1
PDE8B
UNC5C
NUMB
PDE5A
KRT38
CDC23
ABCC3
NOL4
CTSF
TNFSF9
ADAM21
MPDZ
SIGLEC5
FBP2
FPGT
TNFRSF10D
DCAF5
FGF17
FGF16
LIN7A
NRP1
WISP1
ST3GAL5
VNN2
SQSTM1
CCNA1
ENDOU
BSN
AP3D1
HSPB3
SELENBP1
PGLYRP1
KALRN
CLIC3
HIP1R
PKD2L1
CLDN1
BPY2

TBX19
LATS1
SLC16A6
SLC16A4
ARHGEF1
SYNGR3
DYRK1B
CTDP1
OSMR
ARHGEF2
REPS2
SLC33A1
MTA2
TIAF1
MAGI1
CCPG1
GCNT3
GPR50
DHRS3
TSPOAP1
CYTH3
CYTH1
GPR37L1
TAAR2
MMP20
NDST3
RPL23
CPNE6
KL
SLC9A3R1
CD101
TMPRSS11D
FADS2
CYP7B1
EIF2AK3
THEMIS2
CABP1
SLC4A8
MYOT
RPH3AL
GDF15
PTGES
RAB3D
H6PD
NR1D1
SOX13
AKAP12
OR1R1P

PDIA4
VPS9D1
AATK
GNA14
SEC24C
SH3PXD2A
LPIN2
MARF1
SDC3
DEPDC5
GREB1
RAPGEF2
EDEM1
ULK2
PCDHGA8
HERPUD1
GPRIN2
TBKBP1
LZTS3
TMEM94
RNF144A
PHYHIP
CUL7
TESPA1
GAB2
ZNF518A
MED24
PJA2
URB1
ZC3H11A
TRANK1
RHOBTB1
SMG7
PLPPR4
SNAP91
FIG4
SLC35E2
TMCC2
MAFB
DOPEY2
NAALAD2
ACOT8
HDAC6
SH2B3
INSL5
SH2D3C
SH2D3A
IL18BP

PTPRU
ATP9A
USH1C
KCNK7
TSPAN5
CTDSP2
RASGRP1
DNAL4
OPTN
MBNL2
PLXNC1
MSLN
LRRC17
SLC17A2
UBE4B
DSCR3
B3GNT3
CCL26
TRDN
TRIM22
ABCA7
ABCA8
CITED2
BPNT1
BTN3A3
CORO2B
PRG3
ATP8A1
NDRG1
RAPGEF3
ZER1
LRRC41
VAT1
SEMA4B
FBLN5
HYOU1
P3H3
UBD
AGR2
SLC34A2
TACC2
COLEC10
SLCO1B1
TRIM3
RBCK1
TRIM16
RASL10A
IGF2BP2

SCGB1D1
CD226
FUT9
SLC12A7
CHL1
KDM5B
SLC17A3
VAMP5
CYSLTR1
CCR9
GJB6
SDCCAG8
FTCD
PPP1R17
HCST
CD300C
FGL2
ACTL7A
PNPLA6
UTS2
MAGED2
SMPDL3A
PNRC1
LILRB5
SPINK5
LILRB4
KLK11
LILRA1
LILRA3
ZPBP
ABHD2
CNMD
C10orf10
RAPGEF4
KERA
ATE1
BTN3A1
BTN2A1
PTPRT
CD160
CAPN11
SLC7A9
TBC1D8
FICD
TREH
WIF1
FZD10
AKAP13

GALNT5
RASSF8
CA5B
FILIP1L
PXMP4
KLF8
POU6F2
MGAT4A
TMC6
SCRG1
CLCA4
COPG1
RASA3
NLGN4Y
RNF44
PPM1E
VASH1
LMTK2
NLRP1
INPP5F
ZBTB1
DENND3
EPN2
KLRK1
MMRN1
ATF6
DKK1
NINL
DIP2C
MAST1
SORCS3
DAAM1
GOLGA8A
KDM4B
ZNF292
PDZD2
MYT1L
TNIK
SETX
CMTR1
KDM4C
TTLL5
CDK19
DDN
CUL9
ATP10B
COLGALT2
PLXND1

KDM6B
FRMD4B
NCDN
LPIN1
DIP2A
KANK1
MLC1
RPRD2
DNMBP
MGA
POFUT2
KIF13B
UBR2
SIN3B
DNAJC13
TRIM2
WASHC4
SASH1
VPS39
DOCK9
VPS8
FAM189A1
TNS2
SIRT5
KCNH4
KCNH3
MLYCD
RHOQ
CELA3B
ABCB9
ABCA5
HEY1
NPTXR
QPRT
MACF1
DAAM2
BICRAL
KAT6B
ARHGAP45
NNT
CDK20
TSPAN15
PIGN
WBP2
WBP1
CLDN14
PADI4
CDC42EP4

CASP14
LEMD3
DAPK2
SH3BP1
SPO11
KCNE5
CA14
PLD3
TRIM29
TMEFF2
SDF2L1
MAFF
FKBP8
APOL2
HYPM
PRPF40B
SUN2
FSCN2
LMOD1
SPDEF
TTLL1
POM121L1P
HECTD1
POU2F3
RAB26
ABTB2
FAM149A
WHRN
CHRDL2
PLEKHG4
SOSTDC1
WWTR1
C20orf194
SNED1
IBTK
CLIP3
C2CD3
TENM4
LRP10
PLEKHG3
LTN1
CCDC69
TANC2
ABCA12
GIMAP2
RGS22
OR1C1
OR2L2

SLC13A4
EHF
GAPDHS
OR7A17
OR5E1P
LHX6
NUPR1
SLC17A5
HEYL
IL36RN
OR11A1
OR10D1P
SRPK3
OR8B2
OR2W1
OR1J2
GAGE2E
HAVCR1
RNU6-50P
RNU1-1
NBEA
FOXD3
ZBTB32
PELP1
LYPD3
CACNG4
DKK4
DKK2
TJP3
CPAMD8
GLS2
IL37
GPR82
OXGR1
GNMT
GPR162
BBS9
PDCD4
BHLHE22
RPS6KA6
SLCO4A1
CDH20
IGHV1-69
CDH19
DLL1
TRDC
TRBV7-3
TRAV39

TRAV23DV6
TRAV22
TRAV12-2
TRAV9-2
IGLV3-10
IGLV1-47
IGLV1-44
IGLV1-36
IGLC7
REM1
SLC27A6
SLC6A16
C11orf54
IFT81
RGCC
SCG3
ANKRD11
TMOD4
VPREB3
UBN1
NPC1L1
MYLPF
SEC61A1
DMGDH
SLC39A2
BAZ2B
LMCD1
TLX3
PLA2G2E
CRNN
PODXL2
IL22
ARHGEF4
ARHGEF3
SLC45A1
KCNK4
DHH
VILL
CDON
SHANK1
TBX22
SOST
ASCC1
ZBTB7B
PLEKHA8P1
ATL1
YBX2
PLLP

ASB3
INSIG2
ING4
CERCAM
TCEAL9
PLCE1
DRICH1
KLF3
CEND1
RXFP3
BPIFA1
GCNT4
CYP39A1
KCNK9
FAM198B
KRCC1
PHF21A
SPTBN5
ZNF771
DACT1
KLRF1
ANGPT4
POLK
SNX9
PCYOX1
GULP1
HSD17B7
SIRT7
NSG2
YPEL5
RASD1
FKBP7
ASB1
ASB4
ASB2
ACSL5
EMCN
ERAP1
TMEM8B
SYT17
CALML5
TUBA8
ZFYVE1
LRP1B
TPCN1
FXYD4
FGFRL1
GPR87

CDHR5
RAB4B
SLC37A1
PCBP3
FAM3B
SOX18
CYTL1
SLC38A2
MOV10L1
CCSER2
XRN1
ANKIB1
HEATR5B
RBM47
ROBO4
RNF186
SCAND2P
EGLN1
LZTFL1
MXRA8
MINDY2
MANSC1
PDP1
BTN2A3P
XAF1
IL17RD
KLHDC4
PCSK4
TET2
TRPM4
DCHS2
KLHL24
GIPC2
KLHL28
ZNF280D
SWT1
CDHR2
ASPN
NUP62CL
WBP1L
MFSD6
ESRP1
TBC1D8B
RNF43
RASIP1
EXD3
CT55
FKBP14

MOCOS
CRYBG2
SPATA6L
SLC52A1
SOBP
CXorf57
CCDC186
SLC38A4
RALGPS2
WDR60
ANKZF1
C19orf73
DNAAF2
KLHL11
KIAA1551
EXD2
KIRREL1
ZNF654
TMEM140
LRRC36
RNF121
SLC29A3
FBXL8
SLC22A15
TMEM63B
PPP4R1L
ASIC4
ZNF821
DNAH3
KIF27
PPP1R9A
ASAP3
G2E3
TBC1D22B
BTBD2
ZNF334
EDEM2
OGDHL
DHX32
IFT122
C1orf106
ZNF83
MBD5
LMBRD1
SCN3B
ST6GALNAC1
TRERF1
GSDMB

GABRQ
LMO3
GPRC5C
ACSS2
KMT2E
NXF3
KLHL4
PCDHGC5
PCDHGC4
PCDHGA10
PCDHGA2
PCDHGA1
PCDHB15
PCDHB10
TEX13B
ANKH
FAM214A
KIAA1217
C21orf62
CRTAM
LRRC8A
CABP5
EIF4ENIF1
LINC00470
FMN2
TCEAL7
UGGT1
AGPAT3
AGPAT4
DPYSL5
NDUFA4L2
OTOR
MEIS3
GPR108
OLFML3
LHX9
PRDM11
BBX
ACKR3
SLC17A7
PLSCR4
PARP11
CD177
ADCK1
TMEM159
PELI2
SALL4
ZMIZ1

FAM219B
RALGAPA2
ATP10A
ATP8B2
ATP10D
VANGL2
PBXIP1
GALNT16
TMCC3
PPM1H
HHATL
SHROOM4
PLEKHG1
MTUS1
CASKIN2
KIAA1328
NCEH1
ARRDC3
KLHL14
CARNS1
DOCK6
KIF17
CCDC191
FAM135A
TMEM181
ARHGAP21
PDZD4
PITPNM2
FAM234B
SHROOM3
KLHL1
LRCH2
LRRN1
CCDC146
KIAA1522
UVSSA
USP29
PLEKHA4
RNF213
VAT1L
CPNE5
WDFY4
WDR19
ZFYVE28
TRIB3
MARK4
POLD4
GRHL3

SLC46A2
NEUROD4
CXCL16
PLEKHB1
NLRC4
INIP
KMT2C
DNASE2B
DMRT3
RRAGD
TMEM8A
TLNRD1
NTN4
SIGIRR
TRPV4
LGR6
BACH2
HAPLN2
ABHD4
UBE2O
TP53AIP1
ANO3
TSPYL2
LHPP
POPDC2
SMOC2
EPB41L4A
LRRC4
NECAB1
P3H1
EDDM3B
ABCG8
ARHGEF28
SOX17
NFKBIZ
CHST8
RGS18
TEKT3
ALDH8A1
VPS16
ACBD3
PCED1A
IL25
SPATA20
CDCP1
LPIN3
AGXT2
LRRC19

TMEM135
MARCKSL1
PRAMEF1
PRAMEF2
ZBTB10
OTUB2
AHNAK
TRPM8
ELOVL6
SLC25A23
ALG12
CHAC1
LY6G6E
PLEKHF1
PRR15L
MMEL1
BHLHE41
B3GNT4
ZBED2
NKAIN1
CORO7
PGBD5
ACSS3
HMBOX1
SH3TC2
TMEM53
MCPH1
RHBDF2
NEIL1
ZCCHC6
VTCN1
ZDHHC14
GAL3ST4
GALNT12
NPEPL1
WDR59
SH3D21
KCTD17
TBC1D17
ADGB
RERGL
CLMN
CATSPERB
AGBL2
FAM124B
ZDHHC11
THSD4
DCAKD

KIAA0319L
CNTD2
CNTNAP3
ERMP1
PAQR6
ZNF442
FRMD1
DNAJB14
PLEKHH3
ANKRD53
PGAP1
C7orf69
ZFP2
C3orf36
MROH9
ZC3H12A
PGGHG
SPSB1
MYCT1
RUBCNL
ACSF2
PREX2
FAM214B
HSD3B7
ABTB1
PABPC1L
PNPLA3
DCAF11
LPAL2
CD276
ESX1
VWA7
MPIG6B
DUSP16
GRIP2
KDM7A
ZNRD1ASP
SLC2A10
DCSTAMP
FAM49A
MAGED4B
COL21A1
TMEM163
UNC93B1
DEFB126
ADAMTS12
SBF2
KRTAP4-6

ARPC5L
FCRL4
INO80B
SLC25A31
CHST9
RTBDN
COG3
AKR1E2
RASSF5
TEX101
FAM167A-AS1
DYNLRB2
TEKT1
MS4A8
CD99L2
NRIP2
YPEL3
INHBE
SLC25A18
TMEM120A
HMCN1
SPATA9
TTC29
KRTAP3-2
KRTAP9-8
TMPRSS13
B3GNT5
FAM160A2
SLC10A7
ARMC2
FSCB
USP44
C4orf17
RPF2
KIAA1109
LOXL4
SLA2
CHD6
C2orf16
FLYWCH1
LZTS2
SYVN1
ZMAT1
MEGF11
FAM120B
CNFN
MCHR2
MAP1LC3A

LYZL1
KRTAP4-4
NT5C1A
FNDC1
SPINK7
ADGRE3
TRIM63
SLC9A7
INSM2
C9orf24
MS4A14
CAPS2
BEX2
CNDP1
PYROXD2
FCRLA
HPDL
ZNF503
TMEM25
RSPO3
PARP10
ZNF341
TMEM87B
FIZ1
FIBCD1
HSH2D
TNS4
CGNL1
MPND
SYTL1
IGSF21
TOX2
DISP1
RERG
KRTAP4-2
KRTAP3-3
PAQR8
ABCC11
KIAA1644
TUBGCP6
RGS8
SLC22A16
ITPRIP
SCIN
TSLP
LMLN
CHRFAM7A
FGD3

FCHSD1
ATG16L2
SEC16B
AQP10
KLC4
CCDC120
EMILIN3
CEACAM21
TGIF2LX
THAP3
DUOXA1
TGIF2LY
ZNF598
IL33
ESAM
ZNF251
KIFC2
FMNL3
ANKRD30A
ANKRD44
PLXNA4
SYT12
C11orf52
SNX29
RCSD1
LMBRD2
ZNF461
CAPN13
TMEM132C
SCGB3A1
CRB3
PXYLP1
FAM114A1
PKHD1L1
NEURL3
ORAI3
CACNA2D4
TBCK
CADPS2
TTYH2
HTRA3
LENG9
ARHGAP12
RP1L1
FOXQ1
GNG8
TP53INP1
TBC1D27

KRT71
PTH2
GLCCI1
CMTM1
KLHDC7B
LINC00313
LINC00334
TRIM9
MAL2
CYGB
BTBD9
XKR4
KLHL32
SLITRK1
RNF157
C1QTNF1
VASN
SLC26A7
PCMTD1
UHRF2
ZNF689
C12orf56
CTHRC1
BATF2
CYYR1
ACSM1
LRG1
WFIKKN1
MRGPRX3
DEFB118
ANTXR2
C10orf71
MSS51
CFAP70
ANKRD22
LYZL2
CALHM3
PNLIPRP3
TMEM45B
CCDC38
ZNF641
SPIC
AK7
C14orf28
CLBA1
PLD4
ADSSL1
KLHDC1

ACOT4
C16orf46
CMTM3
ZG16B
ZPBP2
C17orf64
SPACA3
ZFP3
SPNS2
EFCAB13
RN7SK
SLC5A10
NLRP13
MOB3A
MISP
CYP4F22
KDF1
CCSAP
DMBX1
PODN
GJB4
DCST2
PIFO
VSTM2L
C20orf85
C20orf141
SLX4IP
CST9L
XIRP2
TAF8
RFTN2
PLEKHH2
SPATA3
TMEM198
TMEM178A
CPO
CPNE4
KCNH8
ZPLD1
ZDHHC19
COL6A6
RTP1
C3orf49
SNTN
SYNPR
LSMEM2
TMEM155
SPATA18

CPEB2
IL31RA
EGFLAM
JMY
ZNF474
RIPPLY2
SRSF12
C6orf141
TRIM40
TRIM50
NOBOX
OR6B1
C7orf31
ZNF572
GOT1L1
DCAF4L2
RNF183
PIP5KL1
ADGRG4
GAB3
MYO3B
DEFB104A
ZFP28
ACTRT2
GATA5
C20orf96
WFDC3
CBLN4
TRIM69
MROH8
UBE2F
KRT72
NEURL2
LINC00266-1
SCP2D1
C20orf173
RIPOR3
R3HDML
MIB2
RAB40A
DYDC1
HECTD2
FGFBP3
XRRA1
MUC15
SESN3
PIWIL4
PLEKHA7

AMDHD1
C12orf60
RAD9B
FAM216B
PPP1R36
C14orf37
ABHD12B
C15orf32
PRR35
GSG1L
ZNF597
C16orf71
CCDC42
C17orf50
TMC8
STARD6
ANKRD29
C19orf18
LYPD4
TMEM190
ZNF563
ZNF558
DIRAS1
CNIH3
SHISA4
ADIG
GTSF1L
HORMAD2
ENTHD1
TEKT4
SMYD1
PUS10
GPBAR1
PPM1L
BTLA
RNF38
ZCWPW2
C3orf30
ZNF827
JAKMIP1
C4orf45
IRX2
TTC23L
CCDC112
PLAC8L1
SLC2A12
PNLDC1
NKAIN2

KHDC3L
LINC01600
BVES-AS1
CRYGN
AGR3
PRAG1
PEBP4
VPS13B
SLC7A13
MCMDC2
NXNL2
HSD17B7P2
TTC39B
FREM1
C9orf84
TSTD2
FAAH2
ARHGAP36
ZDHHC15
RBMY1F
CFAP58
TMTC2
GRASP
SYNE3
CLEC14A
TMEM30B
MDGA2
ODF3L1
MFSD6L
SLFN5
SPPL2C
ZNF579
PLPPR5
FNDC7
TDRD5
CALML6
HFM1
APCDD1L
WBP2NL
LONRF2
TOGARAM2
PARP15
XIRP1
PRICKLE2
RASSF6
RBM46
LCA5
OLIG3

GALNTL5
ZNF467
OLFML2A
ARX
FUT11
S100Z
PSORS1C1
ADAMTS16
SYNPO2
AGO4
SDR16C5
CCER1
LINC01599
FAM227B
TTC21A
NLRP7
FYB2
TVP23C
SPEM2
UNC13D
ZBTB7C
HACD2
SMIM14
TAPT1
KHDRBS2
C7orf33
TSNARE1
HTRA4
ADAM32
SUSD3
NRK
SLC44A5
SMTNL1
ZNF804B
ZCCHC24
STOX1
TBATA
SLC37A2
OOSP2
DOK6
OTUD1
TIGD3
RASGEF1A
REEP3
UCMA
SLC39A12
LINC00305
ADGRF5

C6orf223
RSPH9
TMEM217
FGD2
ARMC12
SYCP2L
PXDC1
BRAT1
SRRM3
KIAA1324L
FAM83B
UNC5CL
ZSCAN23
TMEM130
SPDYA
MS4A6E
DEFB106A
DEFB123
ATP6V0D2
GAS2L2
IL27
RICTOR
IPMK
SLC25A30
EHBP1L1
SNX32
CXorf58
CALHM5
CNIH2
TBC1D28
TMEM86B
TAC4
TCP11L2
RASGEF1C
SYT14
COL6A5
CDRT15L2
NAPSB
CCDC110
NUTM1
HEATR9
RNF214
KLHL34
MEIS3P2
WFDC11
SEC14L3
OR51B5
OR8G7P

JAKMIP3
OR8D2
P4HA3
KLHL35
OTOGL
DPY19L2
HECTD4
C12orf40
SLC46A3
C14orf178
C16orf54
ZNF843
HID1
VMO1
LINC00324
CDRT4
C17orf78
LGALS9B
MIR7-3HG
SLC25A41
RIIAD1
RSPO1
C20orf197
LINC00319
SH2D6
CCDC141
DNAJC5G
IGSF10
SH3PXD2B
KIAA0825
LINC00174
LSMEM1
XKR6
ZNF252P
C8orf31
ZFP41
SCARA5
CRB2
TUSC5
TTC6
RAB37
FAM151A
NLRP10
NLRP14
PRSS53
KLHL17
ZFP69
TEX33

H1FX-AS1
ARSI
TREML3P
RSPO2
BEX5
GOLGA6A
ZKSCAN2
SMTNL2
PRAMEF10
KCNT2
RSPO4
RSPH4A
ZFP57
SERINC2
FAM131C
WDR86
DNAAF3
GATS
C6orf58
SLCO4C1
KAAG1
POLN
LILRA5
ASPG
EFCAB5
ZNF829
ZNF568
B3GNT8
TEX38
RBM43
TRIM73
ENHO
ERCC6L2
SLC27A1
TRIM74
RBMY1D
KRTAP10-4
KRTAP10-6
KRTAP10-2
KRTAP10-12
SLC6A10P
CEP85L
ZNF322P1
GPR153
C10orf99
CC2D2B
TEX36
FAM99A

INSC
GLTPD2
SCIMP
C17orf100
LRRC75A
C17orf98
CCDC103
YPEL2
BTBD17
ZNF790
FLG2
FMO6P
C2orf81
ARHGEF37
IYD
PAGE2B
ZCCHC13
OR8B3
PRAMEF17
ANKRD20A11P
MYO18A
SHC4
SIGLEC16
PRAMEF13
DEFB132
LINC00487
RGPD1
RGS7BP
LCNL1
XKRX
IGLON5
APOBEC4
CUEDC1
MIR124-2
MIR143
MIR192
MIR194-2
MIR30C2
MIR34C
FNDC9
DENND6B
STX19
TMEM41B
CCDC196
CCDC88C
GOLGA6L9
TTLL13P
CTRB2

ODF3B
OR2J1
OR2J3
FAM47C
BPY2B
BPY2C
IGIP
DEFB104B
DEFB106B
LINC00862
C1orf134
TWF1P1
ACOT6
FAM110C
AKAIN1
FAM196A
GRIK1-AS1
ZC3H11B
ZNF862
WASF4P
C15orf56
LINGO3
ADORA2A-AS1
HRCT1
C6orf132
SRGAP2C
RGPD3
GOLGA6C
LGALS9C
LINC01545
RGPD8
MUC5B
MROH1
NUTM2A
NUTM2D
MAGED4
OPN1MW2
CNTNAP3B
CCR2
NUTM2B
PLIN4
GAGE2D
GAGE12B
GAGE12E
GAGE12H
PRAMEF14
RGPD2
SNORD114-10

MIR769
TVP23A
SNORD116-21
TMEM170B
SRRM2-AS1
FAM236A
HDHD5-AS1
LRRC70
TSTD1
CD300LD
GAGE12D
C17orf99
RASA4B
DEFB4B
ZNF605
TRPC5OS
CCDC180
LRRC3C
OCLN
FPGT-TNNI3K
FAM187A
PRORY
RNU6-82P
PYURF
CASP1
RUNX1T1
CNN3
SLC29A1
MTF1
NFATC3
OPRM1
PKN2
EIF2AK2
TRPS1
PCGF2
FXR1
COIL
SMC1A
GAS7
RNMT
USP3
COQ7
TNFSF13B
COPS8
PRRC2C
ATP11A
ZNF107
TBC1D13

ZCCHC2
ZWILCH
TMEM248
CDC42BPG
CDKN2AIP
SCYL3
JPH3
SNX16
PAPD5
VASH2
ACTR5
LNPK
HOPX
PHF5A
PHLDB2
ZNF468
ZNF622
G6PC3
SDR42E1
TRABD2A
ZNF578
C1orf210
ITPRIPL1
GDF7
IBA57
FAM13C
RD3
PLEKHG7
TMEM236
ACTA1
AK4
ANK1
BACH1
CALU
DNA2
ARID3A
DUSP1
E2F6
EIF4EBP1
GLO1
GOLGA4
HNRNPC
MAGEA3
MAGEA6
MAGEA12
NAP1L1
NUCB2
OPRL1

PAFAH2
PBX2P1
PBX2
PMAIP1
POLR2D
PRIM2
RDX
RFC2
MRPL12
RRM2
ATXN2
SOD2
STK4
SURF4
THBS1
TRAPPC10
SUMO1
ZNF200
MLLT10
KMT2D
HIST1H2AK
CLDN12
VAMP3
HAND1
ONECUT2
ATP6V1G1
KIAA0391
EIF4A3
ZNF443
AKAP8
IGF2BP3
MCF2L2
PMPCA
KHNYN
RBFOX2
ARL5A
LATS2
SALL3
THYN1
RNFT1
PLEKHO1
CPA4
RWDD1
CDKAL1
SEMA4C
PARP16
USP47
SLC38A7

YOD1
SOX6
CDV3
ECHDC1
DIABLO
ATXN7L3
RAB25
CGN
PDP2
ARHGAP22
AIDA
PLEKHA3
PHACTR4
ATG9A
ADIPOR2
SLC19A3
C1orf21
ZNF611
FAM103A1
HASPIN
EMILIN2
ZNF644
FUT10
FBXL20
HIST1H2BK
ZNF799
ZNF526
MSI2
PM20D2
ZNF280B
ZNF417
ZNF738
DTX3L
TMTC3
SDE2
KLHDC8B
TMED4
KCTD21
ZBTB8OS
ZNF774
NDUFA4P1
NHLRC2
MALAT1
ATXN7L3B
AMFR
ZFP36L1
CSNK1A1
H1F0

LOXL2
LPP
PRKAG1
PRPS1
PTPN4
RAP2A
SRSF4
SMARCE1
CSDE1
CDK13
URI1
MTMR4
PDIA6
POLI
IKZF2
TOGARAM1
SYNE2
USP22
FCF1
ZFR
FBXL19
ALKBH5
POF1B
RWDD2A
SDR9C7
ZNF513
APOOL
E2F7
PPM1K
PROSER2
EYS
SNX19
ARF5
SCARB2
EIF2B1
GSTM5
KLRD1
MAP4
MRE11
POLR2E
WIPF1
DPM2
KIAA0319
ABI2
PPP6R1
CSTF2T
AGTPBP1
WIZ

WIPF2
DUSP18
ZNF621
GOLGA6L4
GOLGA6L10
ADCY2
ADH4
ALOX5AP
BNIP2
CAPZA1
SIGLEC6
AP1S1
DUSP3
ELF4
ELK1
F2
FANCC
GRK4
GSTM3
GTF2F1
HAS3
HLA-E
IDS
IL2RA
KCNA7
KPNA4
LIMS1
MAT2A
MCAM
MGAT1
MT1A
MT1E
MTAP
MYH2
RPL10A
NFX1
PTPN2
PEX2
RAD21
RBL1
RBMS2
TSPAN31
SCO1
ST3GAL1
ST3GAL2
STXBP2
TEP1
TFDP1

TFDP2
THRA
TIAL1
UBE2G2
UROS
CNBP
ZNF74
ZNF85
ZNF138
ZNF154
ZNF226
ARHGEF5
DGKE
TNFSF14
CDKL1
TAX1BP1
SEC22C
RPS6KA5
BCL7B
MED7
AKAP5
TBPL1
MTFR1
LAPTM4A
BMS1
TOMM70
TECPR2
ZBTB5
HS3ST1
WASF2
PRMT3
ACAA2
SEC23B
ENOX2
YME1L1
MAP3K2
TRAF3IP2
RAB10
HNRNPUL1
BTN3A2
ZNF507
UNC13A
SIK2
WWC1
MGRN1
PPWD1
TNPO3
DNPEP

HEBP2
KCNE4
C22orf24
MTO1
NDUFAF3
KBTBD2
GLCE
PHGDH
BLOC1S6
SULT1B1
COMMD9
MYLIP
RACGAP1
OLA1
EEF2K
AK3
MEMO1
GLRX5
ZNF117
HDAC7
ESF1
MRPS23
MSRB1
TAS2R5
INO80
PAQR5
RHBDL2
PTCD3
PLEKHB2
RBM23
SBNO1
TMEM40
CHDH
NCBP3
NAGK
VEZT
OTUB1
NPLOC4
DEPDC1B
DCP1A
EMC3
ZC3H15
ALG1
KCMF1
LYRM4
JPH2
ERGIC1
CLK4

KIAA1143
XPO5
GNB4
MRPL17
XPNPEP3
NPAS3
TMEM168
ZDHHC6
MRPS25
KRI1
MRPL57
CENPM
ZNF655
TMEM109
TMEM43
MFSD13A
SNX22
SYNPO2L
YIPF5
TSPAN14
TIGD6
RNF170
NETO2
RNF146
THAP2
SYT15
RSPH3
GINS4
C15orf48
MSANTD4
ZNF347
LRCH3
PLXDC2
CEP89
TMEM250
ANGEL2
SLC25A46
YTHDC1
MFSD4B
SPECC1
ESCO1
SMYD4
FAM210B
MOGAT1
LEAP2
KRBA2
FBXO27
TCEANC2

FITM2
TSHZ2
ENPP6
MPLKIP
ASB6
STK35
KDELC2
TMEM120B
ZSCAN29
BROX
TMEM56
BTBD19
GPR155
SGO1
ZMAT2
BMT2
RDH10
PGBD4
ZNF320
DENND2C
PGAM5
ALG14
RAB12
SLC36A1
AKR7L
ZNF620
GK5
NKPD1
ZNF841
VSIG1
FMN1
MOGAT3
DNAJB13
C3orf62
VWC2
RGS9BP
ZNF805
AGMO
CISD2
FAM229B
AGAP9
RNF222
ZBTB8A
POM121L7P
TRIM23
ZFP36L2
BUB1
CDC5L

GGCX
GNL1
RPS24
TPM1
STK38
PPIP5K2
ANGPTL3
DESI1
DNAJC10
TMEM106B
ACER3
CADM3
HIVEP3
ZSCAN16
SLC38A9
C8orf37
POTED
SIGLEC14
ATP1B3
B2M
BMI1
CAPZA2
UBE2K
FOXN2
NRF1
PPP2R1B
SKP1
TJP1
SOCS1
COL4A3BP
SCML2
RABAC1
GNA13
SAMD4A
OTUD3
BRD4
ATRNL1
ARRDC2
MMADHC
RSL24D1
AZIN1
GIN1
FBXW7
FEM1A
ACTR3B
SIKE1
FSD1L
FYTTD1

CCDC74A
CCDC74B
NXPE3
FNIP1
MYL12B
C5orf24
MIPOL1
ZNF449
JAZF1
RNF144B
NUDT7
PTAR1
NOTCH2NL
ACBD7
ACTR3C
FAM182B
COMMD3-BMI1
ADCYAP1
GRK2
CRISP1
CACNA1E
ENTPD5
FOXN3
VCAN
DLX3
ETV6
FANCF
FOXF2
FZD2
GABRB2
GALR1
HLA-DRB1
HRH1
HSPA6
HSPD1
EIF3E
LBR
NFIA
NINJ1
PAX5
PAX6
PCSK2
PKHD1
PLCG1
POU3F2
PPP2R1A
RAB3B
RPS21

SRSF1
SHMT1
SPAST
SPN
AKR1D1
SSB
SSR3
STAT5B
TBCA
TNFAIP3
UQCRFS1
ZFX
ZNF18
ZNF24
ZNF124
ZNF134
ZNF225
TTF2
CADPS
SAP30
CDKL2
PAPSS2
SNAP29
MDC1
PCLAF
HS3ST3B1
COX17
ARPC1B
RAD50
ARL4C
TOB1
TENM1
LILRB2
CHERP
ARFGEF2
ZNF268
ADAMTS8
NUPL2
PLA2G16
PDCD10
TFEC
PDCD11
KLHDC10
TMEM131L
FAM208A
UFL1
RASGRP3
APPL1

RSL1D1
CYFIP2
PDE7B
MORC1
SULT1C4
KCNMB4
SLC25A24
SH3KBP1
PDE11A
DERL2
MRPS18C
RSRC1
TAOK3
GSKIP
LUC7L2
CLIC5
MRPL50
DDIT4
VSIG10
ALKBH4
BCOR
SSH3
C1orf56
BSDC1
SLC30A10
VPS50
TSR1
TXLNG
UBAP2
AJAP1
PNO1
GATAD2B
RNF150
PCDH19
MIB1
JCAD
BLOC1S5
IKZF5
RNF128
NDNF
ZMAT4
GTDC1
NIPAL2
CXorf21
COLEC12
STMN4
FAM107B
TMTC1

ZIC4
TMEM107
FRMPD3
NAV2
SLC25A21
MIDN
ZNF616
ZNF160
COX19
FAAP24
OMA1
FBXO17
FGD4
TRAPPC6B
AFMID
DNAJC21
FAM199X
GLIPR1L2
SLC35G3
C1orf52
PHF13
ZNF362
KANSL1L
SGO2
CNKSR3
CAMSAP1
COMMD6
NT5DC1
NEGR1
TAS2R30
ZNF283
TCAIM
SREK1IP1
CCDC39
FAM89A
TMEM81
FBXO47
RNF165
LINC00598
C7orf55-LUC7L2
CHD4
CNN2
COX10
ETS2
F2RL1
KDSR
SLC37A4
GABRA2

GSPT1
HELLS
HMGN2
ID3
MAF
PPARD
SRP72
PPP1R11
DYNLT1
TIMP1
ZNF45
MAP7
ITM2A
HS2ST1
RAB11FIP3
STARD8
EXOG
PTBP3
CNIH1
RCAN2
IGSF6
CACNG2
PDLIM5
ARPP19
FGFR1OP
SYT11
KCTD2
RPL36
CLEC2D
SLC35C2
SH3GLB1
NIN
MBTPS2
NUP54
CNNM2
VPS53
RBM41
ATF7IP
ANKRD50
STRIP2
SORCS2
ALPK3
TRMT5
KLHL12
AZI2
TET1
PUS7L
KATNAL1

SRRM4
FAM104A
NUDCD1
NUS1
ZNF354B
IFFO2
VPS37A
S100A16
PYHIN1
COMMD1
ARL6IP6
FLCN
CNOT6L
ZDHHC24
EOGT
ZNF707
LUZP2
SPOPL
GPR141
FAM19A1
ATP5EP2
ANKRD33B
CTPS1
HES1
NEFL
N4BP1
RALGAPB
CEP97
CABLES1
C11orf84
ARSK
NANOS1
CLCN7
HINT1
MYH9
RFX1
WNT8B
WDTC1
SLCO3A1
NEURL1B
SMOX
ZC2HC1C
URM1
ELMSAN1
ASB16
ZSWIM7
DNAJC19
SIK1

FAM71B
NLRP11
ATXN1L
MTRNR2L7
MTRNR2L3
MTRNR2L11
